# Targeting the LSD1-G9a-ER Stress Pathway as a Novel Therapeutic Strategy for Esophageal Squamous Cell Carcinoma

**DOI:** 10.34133/2022/9814652

**Published:** 2022-06-01

**Authors:** Hongxiao Wang, Zijun Song, Enjun Xie, Junyi Chen, Biyao Tang, Fudi Wang, Junxia Min

**Affiliations:** ^1^The First Affiliated Hospital, The Fourth Affiliated Hospital, Institute of Translational Medicine, School of Public Health, Cancer Center, State Key Laboratory of Experimental Hematology, Zhejiang University School of Medicine, Hangzhou 310058, China; ^2^The First Affiliated Hospital, The Second Affiliated Hospital, Basic Medical Sciences, School of Public Health, School of Pharmaceutical Science, Hengyang Medical School, University of South China, Hengyang 421001, China; ^3^Department of Pathology, Henan Provincial People's Hospital, People's Hospital of Zhengzhou University, People's Hospital of Henan University, Zhengzhou 450003, China

## Abstract

Despite recent advances in the management and treatment of esophageal squamous cell carcinoma (ESCC), the prognosis remains extremely poor, and current nonsurgical treatment options are limited. To identify new therapeutic targets, we screened a curated library of epigenetic compounds using a panel of cancer cell lines and found that coinhibiting the histone demethylase LSD1 and the histone methyltransferase G9a potently suppresses cell growth; similar results were obtained by knocking down both LSD1 and G9a expression. Importantly, we also found that inhibiting LSD1 and G9a significantly decreased tumor growth in a xenograft mouse model with ESCC cell lines. To examine the clinical relevance of these findings, we performed immunohistochemical analyses of microarray profiling data obtained from human esophageal squamous cancer tissues and found that both LSD1 and G9a are upregulated in cancer tissues compared to healthy tissues, and this increased expression was significantly correlated with poor prognosis. Mechanistically, we discovered that inhibiting LSD1 and G9a induces cell death via S-phase arrest and apoptosis, and cotargeting ER stress pathways increased this effect both *in vitro* and *in vivo*. Taken together, these findings provide compelling evidence that targeting LSD1, G9a, and ER stress-related pathways may serve as a viable therapeutic strategy for ESCC.

## 1. Introduction

Esophageal cancer (EC), including esophageal squamous cell carcinoma (ESCC) and esophageal adenocarcinoma (EAC), is one of the most common malignancies, ranking seventh in global incidence and sixth in overall mortality [1]. In cases with locally advanced EC, esophagectomy remains the mainstay treatment, although chemotherapy and radiation therapy have also been recommended as the standard-of-care [2]. Due to limited treatment efficacy and the risk of developing drug resistance to single-agent therapies, combination therapies are emerging as a more effective strategy for treating EC. In the past decade, a handful of targeted therapies have been approved for EC, including trastuzumab combined with chemotherapy as the first-line treatment for HER2-positive esophageal adenocarcinoma [3], the vascular endothelial growth factor (VEGF) receptor inhibitor ramucirumab as the second-line treatment for gastric or gastro-esophageal junction adenocarcinoma [4, 5], and pembrolizumab for the treatment of PD-L1-positive ESCC or EAC [6]. Despite significant advances in the development of these molecular-targeted therapies and immunotherapies, the clinical outcome and survival rate of patients with advanced EC remain low [7, 8]. Therefore, new therapeutic targets are urgently needed in order to develop new therapeutic strategies designed to improve the treatment and management of EC.

Epigenetic modifications refer to covalent modifications to either histone molecules or DNA that are heritable, reversible, and do not alter the DNA sequence. A growing number of studies have highlighted the importance of epigenetic changes in cancer treatment and prognosis [9]. Currently, nine epigenetic agents are available in the US for treating cancer, including two DNA methyltransferase (DNMT) inhibitors (azacitidine and decitabine), four histone deacetylase (HDAC) inhibitors (vorinostat, romidepsin, belinostat, and panobinostat), two isocitrate dehydrogenase inhibitors (enasidenib and ivosidenib), and one EZH2 inhibitor (tazemetostatv) [10]. Despite the success of these agents, current epigenetics-targeted therapies have been limited to hematological malignancies. However, these therapies have high therapeutic potential for various solid tumors based on preclinical and clinical trials, including the DOT1L methyltransferase inhibitor pinometostat and the HDAC6 inhibitors ricolinostat, citarinostat, and KA2507 [10].

In addition, epigenetic changes have long been known to play important roles in the initiation, progression, and metastasis of EC [11, 12]. At the DNA level, epigenetic “writers” (e.g., methyltransferases such as DNMT1 and DNMT3B) and “erasers” such as TET2 and TET3 have been associated with EC [13, 14]. With respect to histone modifications, the increased expression of epigenetic “writers” such as acetyltransferases and methyltransferases (e.g., HAT1, p300, AIB1, SUV39H1, G9a, SMYD3, and EZH2) and “erasers” such as HDAC1-5, SIRT1, SIRT3, SIRT6, LSD1, and KDM5B have been linked to disease progression in EC [15, 16]. In a phase 1 clinical trial, the DNA methyltransferase inhibitor 5-azacitidine was shown to potently increase the sensitivity of resectable esophageal and gastric adenocarcinomas to conventional therapy [17]. Thus, targeting epigenetic changes is a promising new therapeutic strategy for EC.

In China, ESCC accounts for more than 90% of all EC cases. The poor prognosis, limited treatment options, and consistent reports of epigenetic changes associated with EC indicate that novel epigenetic targets should be identified for treating ESCC. Therefore, we performed an epigenetic drug library screening in order to identify potential epigenetic modulators that could be targeted in ESCC. In addition, we functionally validated our results using both pharmacological and genetic approaches, and we performed transcriptomics and tissue microarray analyses using human ESCC cell lines and patient tissue samples, respectively. Finally, we examined whether compounds that target epigenetic modifiers can serve as a potential strategy for treating ESCC.

## 2. Results

### 2.1. Screening of Epigenetic Compounds Reveals That Both LSD1 and G9a Play a Role in ESCC

**Figure 1:**
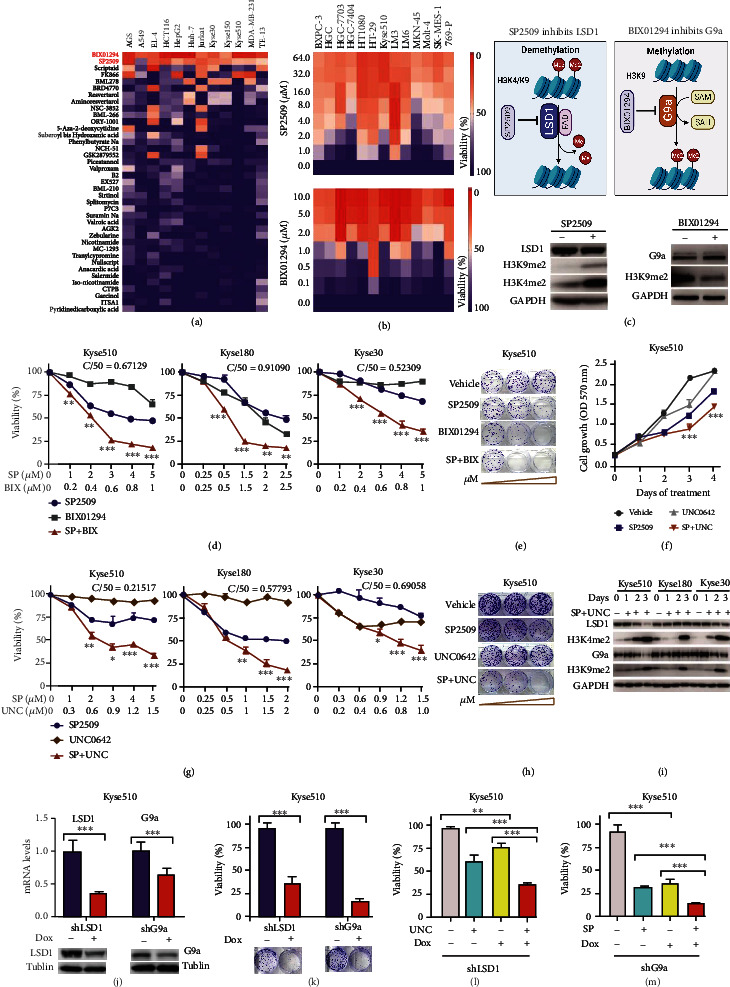
A subset of esophageal squamous cancer cell lines is vulnerable to inhibitors of LSD1 and G9a. (a) Heatmap of cell viability measured in the indicated cancer cell lines treated with the indicated small-molecule epigenetic modifiers (applied at 5 *μ*M each). (b) Heatmap of cell viability measured for the indicated cell lines treated with the indicated concentrations of SP2509 and BIX01294. (c) Model depicting the proposed actions of SP2509 and BIX01294 by inhibiting LSD1 and G9a, respectively. FAD: flavin adenine dinucleotide; SAM: S-adenosyl-L-methionine; SAH: S-adenosyl homocysteine; Me: mono-methylation; Me2: di-methylation. (d) Summary of the viability of the indicated ESCC lines treated with the indicated concentrations of SP2509 (SP) and BIX01294 (BIX). CI50: combination index at median effect of drug treatment. CI50 < 1 indicates synergism. (e) Colony formation assay of Kyse510 ESCCs treated with vehicle or increasing concentrations of SP2509 and/or BIX01294. (f) Time course of cell growth of Kyse510 ESCC cells treated with 4 *μ*M SP2509 and/or 1.8 *μ*M UNC0642 for the indicated number of days. (g, h) Same as (d) and (e), respectively, except the cells were treated with SP2509 and UNC0642 (UNC). (i) ESCC cells were treated with vehicle or 10 *μ*M SP2509 and 10 *μ*M UNC0642 for 0, 1, 2, or 3 days, followed by Western blot analysis. (j) Silencing efficiency of Dox-inducible shRNA constructs targeting LSD1 and G9a was measured at the mRNA and protein levels in Kyse510 cells treated with vehicle or 200 ng/ml Dox for 4 days. (k) Cell viability and colony formation were measured in shRNA-expressing Kyse510 cells treated with vehicle or 200 ng/ml Dox for 6 days and 10 days, respectively. (l, m) Viability of Kyse510 cells stably expressing the Dox-inducible shLSD1 (l) or shG9a (m); where indicated, cells were treated for 3 days with 200 ng/ml Dox and 0.6 *μ*M UNC0642 (l) or 3 *μ*M SP2509 (m). ^∗∗^*p* < 0.01 and ^∗∗∗^*p* < 0.001 (unpaired Student's *t*-test). The data are representative of three independent experiments.

To identify small-molecule compounds that can inhibit epigenetic modifiers in various cancer types, we screened a library of 39 inhibitors (Supplementary Table S1) by treating a panel of cancer cell lines for 3 days and then measuring cell viability. As shown in Figure 1(a), a heatmap of cell viability revealed that SP2509 and BIX01294 were the two most potent compounds, and this finding was confirmed using an additional panel of cancer cell lines, in which both SP2509 and BIX01294 significantly reduced cell viability when applied at ≥5 *μ*M for 3 days (Figure 1(b)). SP2509 is a specific, reversible noncompetitive inhibitor of LSD1, an amine oxidase that demethylates H3K4me1/2 and H3K9me1/2 by binding to the flavin adenine dinucleotide (FAD) pocket [18, 19]. BIX01294, a competitive inhibitor of histone methyltransferase G9a, was discovered by Kubicek et al. using high-throughput screening and exerts its inhibitory effect by binding to the substrate-binding groove in G9a [20]. G9a primarily catalyzes the methylation at Lys9 in histone H3 molecules [21]. A working model depicting how SP2509 and BIX01294 inhibit LSD1 and G9a, respectively, is shown in Figure 1(c). Importantly, Western blot analysis showed that treating cells with SP2509 increased both H3K9me2 and H3K4me2 levels, while treating cells with BIX01294 decreased H3K9me2 levels (Figure 1(c), bottom), consistent with the notion that these compounds inhibit the enzymatic activity of LSD1 and G9a, respectively.

Next, we investigated whether treating cells with both SP2509 and BIX01294 is more effective at reducing cell viability compared to treating cells with each inhibitor separately. We found that cotreating cells with SP2509 and BIX01294 only mildly increased cell death compared to each inhibitor separately in HepG2, Huh7, AGS, Jurkat, A549, and HT1080 cells (Figure S1a). In contrast, this treatment caused significantly more cell death in the ESCC cell lines Kyse510, Kyse180, Kyse30, ECA109, and TE-1 (Figure 1(d) and Figure S1b), and the combination index (*CI*_50_) of SP2509 and BIX01294 calculated via CompuSyn was less than 1, indicating a synergistic effect of SP2509 and BIX01294 on ESCC cells. Moreover, inhibiting both LSD1 and G9a reduced ESCC cell colony formation more than inhibiting either enzyme alone (Figure 1(e)), suggesting that ESCC cells are particularly susceptible to strategies that target both LSD1 and G9a. We therefore focused on studying the combinational inhibitory effect of LSD1 and G9a on ESCC cells in our subsequent experiments. Interestingly, we found that the sensitivity of ESCC cells to LSD1 or G9a inhibitor was not related to the expression levels of either LSD1 or G9a (Figure S1c-d).

BIX01294 is highly toxic; however, the primary epigenetics library was obtained from the National Compound Resource Center which contained only this G9a inhibitor. UNC0642—a recently developed molecule based on the structure of BIX01294—was shown to have lower toxicity, higher selectivity, better lipophilic properties, higher cell membrane permeability, and improved pharmacokinetics in animal models compared to BIX01294 [22]; we therefore used UNC0642 instead of BIX01294 for our subsequent experiments. Consistent with our results obtained using BIX01294, we found that cotreating ESCCs with both SP2509 and UNC0642 caused significantly higher levels of cell death (Figures 1(f) and 1(g) and Figure S1e) and significantly lower colony formation (Figure 1(h)) compared to treating cells with either SP2509 or UNC0642 alone. Moreover, treating the ESCC cell lines with both SP2509 and UNC0642 for 1-3 days increased both the di-methylation levels of Lys4 in histone H3 (H3K4me2) and the di-methylation levels of Lys9 in histone H3 (H3K9me2) (Figure 1(i)), suggesting that these inhibitors' effects on the viability of ESCCs are mediated predominantly by inhibiting LSD1.

### 2.2. Knocking Down Both LSD1 and G9a in ESCC Cells Causes Cell Death

To validate the results obtained by pharmacologically inhibiting LSD1 and G9a, we knocked down LSD1 and G9a expression in ESCC cells using shRNA. We first tested four shRNAs targeting *LSD1* mRNA and four shRNAs targeting *G9a* mRNA. We found that all four *LSD1* shRNAs significantly reduced cell viability (Figure S2a) and eliminated ESCC cell colony formation (Figure S2b). Surprisingly, similar to the synergistic effect of treating cells with both SP2509 and UNC0642, we found that the shLSD1-1 shRNA (which was the most effective *LSD1* shRNA construct at reducing colony formation) significantly increased the potency of UNC0642 for inducing cell death in ESCC cells (Figure S2c). With respect to G9a, we found that knocking down *G9a* mRNA significantly reduced ESCC cell viability and colony formation (Figure S2d-e), and the most effective construct, shG9a-2, significantly increased the ability of SP2509 to induce ESCC cell death (Figure S2f).

Next, we used the same seed sequences contained in the shLSD1-1 and shG9a-2 shRNA constructs to generate doxycycline- (Dox-) inducible shRNA constructs and then used these constructs to create stable Kyse510 and Kyse30 cell lines. Knockdown efficiency of LSD1 and G9a in Kyse510 cells was confirmed at both the mRNA and protein levels (Figure 1(j)). As shown in Figure 1(k), Dox-induced knockdown of either LSD1 or G9a in Kyse510 cells significantly reduced both cell viability and colony formation. Moreover, we found that Dox-induced knockdown of LSD1 combined with UNC0642 treatment caused significantly more cell death compared to either treatment alone (Figure 1(l)); similarly, Dox-induced knockdown of G9a combined with SP2509 treatment also caused significantly more cell death compared to either treatment alone (Figure 1(m)), and similar results were obtained in Kyse30 cells (Figure S2g-i). Taken together, these data suggest that knocking down LSD1 and G9a increases the efficacy of UNC0642 and SP2509, respectively, at inducing death in ESCC cells, thus resembling the effects of pharmacologically inhibiting LSD1 and G9a.

### 2.3. LSD1 and G9a Confer Epigenetic Dependency to ESCC Cells in an *In Vivo* Model

**Figure 2:**
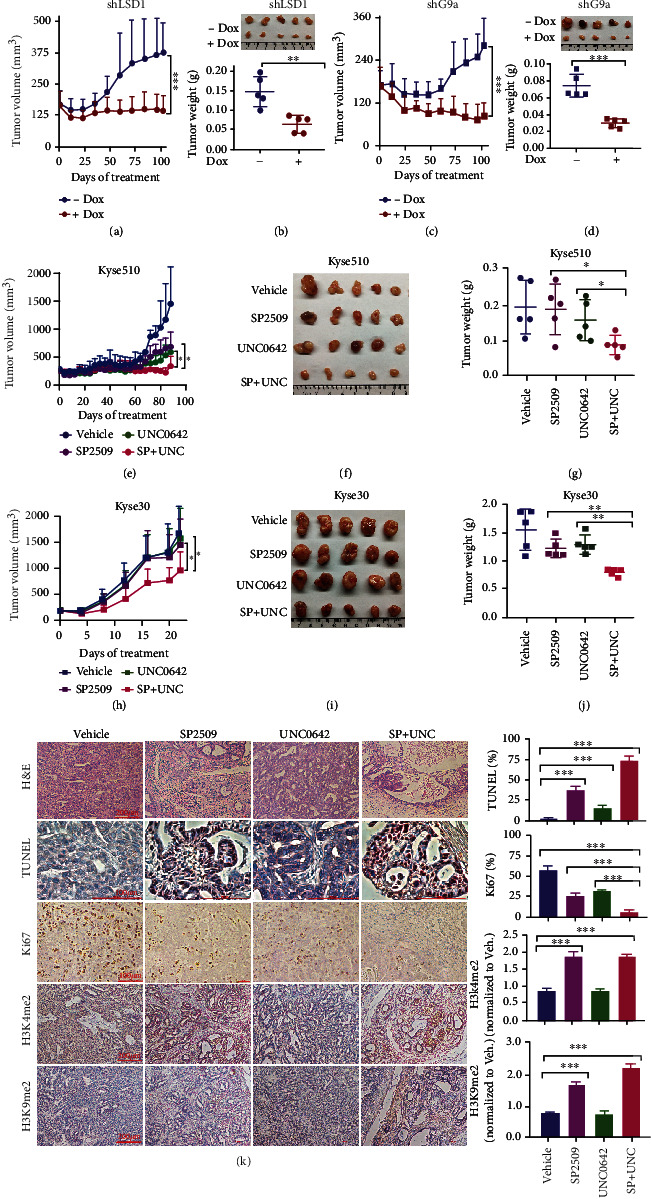
LSD1 and G9a are required for *in vivo* tumor growth in an ESCC xenograft mouse model. (a–d) ESCC cells Kyse510 stably expressing the Dox-inducible shLSD1 (a, b) or shG9a (c, d) constructs were transplanted into mice. The mice were then treated with vehicle (control) or Dox for the indicated number of days, and tumor volume and final tumor weight were measured (*n* = 5 mice/group). (e–j) Kyse510 cells (e–g) or Kyse30 cells (h–j) were transplanted into mice. The mice were then treated with the indicated compounds for the indicated number of days, and tumor volume and final tumor weight were measured (*n* = 5 mice/group). (k) Tumors were obtained from xenograft recipient mice treated as indicated, and sections were stained with hematoxylin and eosin (H&E), TUNEL, Ki67, H3K4me2, and H3K9me2; the percentage of positive cells are shown at the right (*n* = 3 tumors/group). ^∗^*p* < 0.05, ^∗∗^*p* < 0.01, and ^∗∗∗^*p* < 0.001 (unpaired Student's *t*-test).

To examine the *in vivo* effect of genetically reducing LSD1 and/or G9a expression, we used a xenograft mouse model in which Dox-inducible LSD1 or G9a knockdown ESCC cells Kyse510 were transplanted into mice, which were then given either regular drinking water (as a control) or drinking water containing 2 mg/ml Dox to induce shRNA-mediated knockdown in the transplanted ESCC cells. We found that compared to control-treated mice, Dox-induced knockdown of either LSD1 or G9a significantly reduced tumor growth, which was reflected by significantly reduced tumor volume and weight (Figures 2(a)–2(d)), without affecting total body weight (Figure S3a, c). In addition, Dox-induced LSD1 knockdown significantly increased both H3K4me2 and H3K9me2 levels (Figure S3b), whereas Dox-induced G9a knockdown decreased H3K9me2 levels (Figure S3d).

Given the potent, synergistic effect of inhibiting both LSD1 and G9a on the viability of cultured ESCC cells *in vitro*, we next tested whether pharmacologically inhibiting these two enzymes affects the *in vivo* viability of xenografted Kyse510 and Kyse30 ESCCs. We found that cotreating mice with SP2509 and UNC0642 significantly reduced ESCC tumor growth compared to vehicle-treated mice and compared to mice treated with either SP2509 or UNC0642 alone (Figures 2(e)–2(j)), again without affecting total body weight (Figure S3e-f). Histological analyses of Kyse510-derived xenograft tumors revealed robust morphological changes in mice cotreated with SP2509 and UNC0642, with extensive necrosis in the tumor center surrounded by degenerating tumor cells with intermingled nuclear debris (Figure 2(k)). Moreover, TUNEL and Ki67 staining revealed that SP2509 treatment alone, UNC0642 treatment alone, and cotreatment with both SP2509 and UNC0642 significantly increased apoptosis and reduced cell proliferation in the tumors, with the strongest effects measured in the cotreated group (Figure 2(k)). Thus, inhibiting both LSD1 and G9a significantly reduces tumor growth in our ESCC xenograft model.

LSD1 specifically removes methyl groups from H3K4me2 and H3K9me2, whereas G9a primarily catalyzes the methylation of H3K9. Our immunostaining experiments revealed that both H3K4me2 and H3K9me2 levels were significantly higher in tumors of SP2509-treated mice and mice treated with both SP2509 and UNC0642. In contrast, H3K9me2 levels were unchanged in mice treated with UNC0642 alone, possibly due to low basal levels of H3K9me2.

### 2.4. Increased LSD1 and G9a Expression Are Correlated with Poor Prognosis in Patients with ESCC

**Figure 3:**
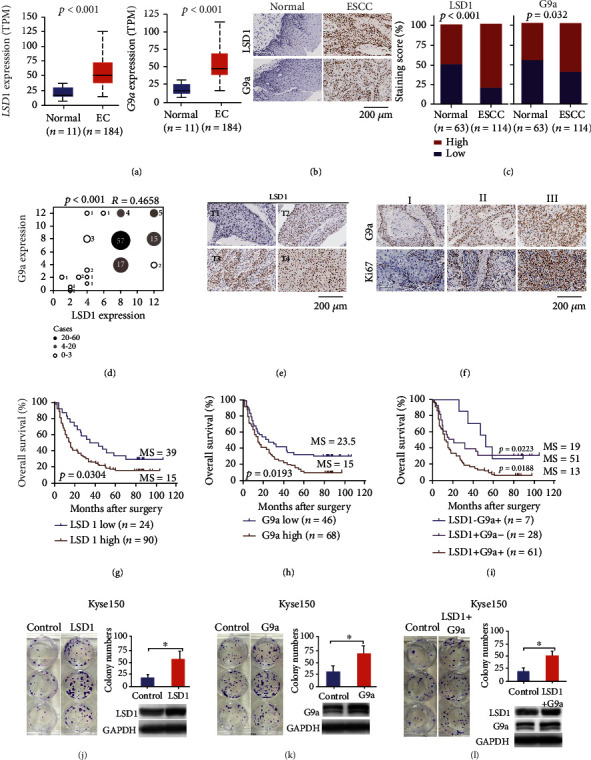
High-expression levels of LSD1 and G9a are associated with poor outcome in patients with ESCC. (a) Summary of *LSD1* and *G9a* mRNA in healthy tissues and tumor tissues obtained from patients with esophageal cancer. (b, c) Representative images (b) and staining score distribution (c) of the indicated tissues immunostained for LSD1 and G9a. (d) Correlation analysis between LSD1 and G9a protein levels in ESCC tissues; the corresponding *p* value and Pearson's correlation coefficient are shown. (e) Representative images of LSD1-stained sections of ESCC samples at the indicated T stage. (f) Representative images of G9a-stained and Ki67-stained sections of ESCC samples with the indicated tumor grade. (g–i) Kaplan–Meier survival curves of 114 patients with ESCC stratified according to tumor LSD1 expression irrespective of G9a expression (g), tumor G9a expression irrespective of LSD1 expression (h), and both LSD1 and G9a expression (i); the pair-wise log-rank test was used to analyze overall survival, and median survival (MS, in months) is shown for each group. (j–l) Colony formation assay of ESCC cells overexpressing LSD1 (j), G9a (k), or both LSD1 and G9a (l), with corresponding representative Western blot analyses. ^∗^*p* < 0.05 (unpaired Student's *t*-test).

To examine whether LSD1 and/or G9a expression is correlated with the clinical and pathophysiological characteristics of EC, we analyzed The Cancer Genome Atlas (TCGA) database via the Ualcan (http://ualcan.path.uab.edu/index.html) and GEPIA (http://gepia.cancer-pku.cn) websites [23, 24]. We found that the mRNA levels of both *LSD1* and *G9a* were significantly higher in EC samples and several other tumor types compared to healthy tissues (Figure 3(a) and Figure S4a-b). When we stratified EC cases into two major histological subtypes (squamous cell carcinoma and adenocarcinoma), we found significantly higher levels of *LSD1* expression in squamous cell carcinomas compared to adenocarcinomas, but no difference in *G9a* expression between these two EC subtypes (Figure S4c). Moreover, we found a significant correlation between *LSD1* mRNA levels and *G9a* mRNA levels in human EC samples (Figure S4d), suggesting that these two genes may be coregulated in these tissues.

Next, we measured LSD1 and G9a protein levels in a tissue microarray consisting of 114 primary EC tissue samples (all derived from esophageal squamous cancer patients) and 63 matched adjacent (control) tissue samples by performing an immunohistochemistry assay; Supplementary Table S2 lists the clinical and pathological features of these 114 patients. As shown in Figures 3(b) and 3(c), both LSD1 and G9a levels were significantly higher in the ESCC samples than in control samples. Specifically, we found high levels of LSD1 protein in 89 out of 114 ESCC samples compared to 31 out of 63 control samples (78% vs. 49%, respectively; *p* < 0.001), and high levels of G9a protein in 68 out of 114 ESCC samples compared to 27 out of 63 control samples (60% vs. 43%, respectively; *p* < 0.05). Moreover, consistent with our mRNA analysis from the TCGA database, we found that LSD1 protein levels were significantly correlated with G9a proteins levels among the ESCC samples (*p* < 0.0001, *R* = 0.4658) (Figure 3(d)). Next, we analyzed the association between LSD1 and/or G9a expression and the pathological features of ESCC patients. As shown in Table 1, LSD1 expression was significantly correlated with patient age and T stage (Figure 3(e)), but not with sex, tumor grade, N stage, TNM stage, or expression of the markers p53, Ki67, PD-L1, or CD8. In contrast, G9a expression was significantly correlated with tumor grade and Ki67 expression (Figure 3(f)), but not with patient age, sex, T stage, N stage, TNM stage, or expression of p53, PD-L1, or CD8.

With respect to patient outcome, Kaplan–Meier survival curves revealed that patients with high tumor LSD1 expression (Figure 3(g)) and patients with high tumor G9a expression (Figure 3(h)) had poorer survival compared to patients with relatively low expression of these proteins. The median survival time of patients with high LSD1 expression—regardless of G9a expression—was 15 months, compared to 39 months for patients with low LSD1 expression (log-rank score: 4.686, *p* = 0.0304). Similarly, the median survival time of patients with high G9a expression—regardless of LSD1 expression—was also 15 months, compared to 23.5 months for patients with low G9a expression (log-rank score: 5.478, *p* = 0.0193). Notably, patients with high expression levels of both LSD1 and G9a had even worse survival, with a median survival time of only 13 months, significantly shorter than patients with high LSD1/low G9a expression (19 months) and patients with high G9a/low LSD1(51 months) expression (Figure 3(i)). Univariate and multivariate analyses also showed that LSD1 and/or G9a protein expression and TNM stage are significantly correlated with overall survival, while univariate analysis shows that the sex, T stage, and N stage are significantly correlated with overall survival (Table S3). To measure whether overexpression of LSD1, G9a, or both affects the *in vitro* growth of ESCCs, we performed a colony formation assay using Kyse150 cells and found that overexpressing LSD1 alone, G9a alone, or both LSD1 and G9a potently increased cell growth (Figures 3(j)–3(l)). Taken together, these clinical and *in vitro* results provide compelling evidence that LSD1 and/or G9a may serve as valuable prognostic markers as well as possible therapeutic targets for ESCC.

### 2.5. Integrative Transcriptomics Analyses of Pharmacological and Genetic Inhibition of LSD1 and G9a in ESCC Cells

**Figure 4:**
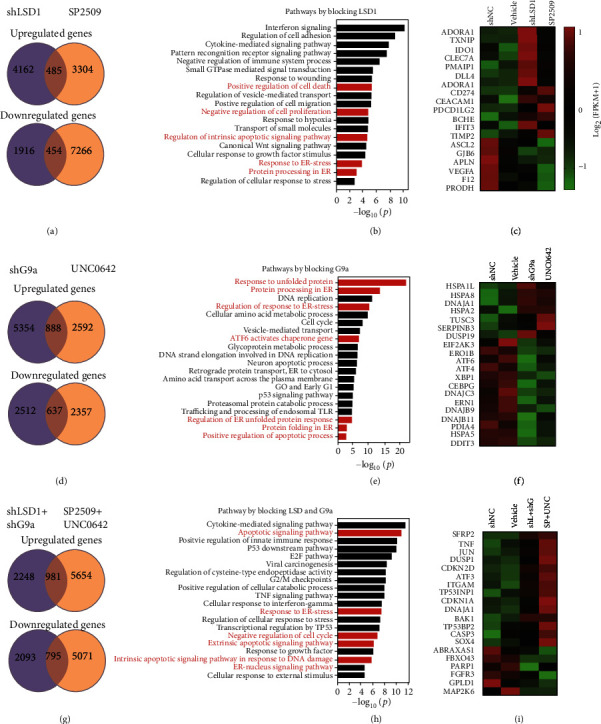
Integrative transcriptomics analyses of ESCC cells following pharmacological or genetic inhibition of LSD1 and G9a. (a) RNA-seq analysis was performed on ESCC cells in which either LSD1 expression was knocked down using shRNA or LSD1 was inhibited with SP2509; differentially expressed genes were determined by comparing with ESCC cells expressing a control nonsilencing shRNA (shNC) and vehicle-treated ESCC cells, respectively, and are summarized using Venn diagrams. (b) Gene Set Enrichment Analysis of the overlapping differentially regulated genes in (a). (c) Heatmap of the differentially expressed genes in the “cell death,” “apoptosis,” and “ER stress” pathways. (d–f) Same as (a–c), except G9a was knocked down using shRNA or inhibited with UNC0642. (g–i) Same as (a–c), except both LSD1 and G9a were knocked down with shRNA or both LSD1 and G9a were inhibited with SP2509 and UNC0642.

Next, we performed global gene expression profiling to investigate the effects of targeting LSD1 and/or G9a in ESCC cells using either pharmacological or genetic approaches combined with RNA sequencing (RNA-seq) analysis. We found that inhibiting LSD1 led to a total of 485 upregulated genes and 454 downregulated genes (defined as *p* < 0.05 and a fold change in either direction of ≥1.3) compared to the corresponding control groups (Figure 4(a)). Gene Set Enrichment Analysis (GSEA) using the web-based portal Metascape (a gene annotation and analysis resource available at https://metascape.org) [25] revealed that genes involved in “positive regulation of cell death,” “negative regulation of cell proliferation,” “apoptosis,” “response to ER stress (endoplasmic reticulum stress),” and “protein processing in ER” were differentially expressed (Figure 4(b)). These differentially regulated genes, which include *ADORA1*, *TXNIP*, *IDO1*, and *ASCL2*, are shown in Figure 4(c). We further analyzed these upregulated and downregulated genes and found that the significantly upregulated pathways involved apoptosis and the release of cytochrome c from mitochondria (Figure S5a), while the significantly downregulated pathways included the response to ER stress, protein folding in the ER, and the IRE1-mediated unfolded protein response (Figure S5b).

In addition, we found that inhibiting G9a led to a total of 888 upregulated genes and 637 downregulated genes (Figure 4(d)). GSEA revealed that genes involved in ER-associated pathways, including “response to unfolded protein,” “protein processing in ER,” “regulation of response to ER stress,” and “ATF6 activates chaperone genes,” were differentially expressed (Figure 4(e)); these genes include *HSPA1L*, *HSPA8*, *EIF2AK3*, *ATF6*, *ATF4*, and *DNAJB9* (Figure 4(f)). The differentially regulated genes in the “response to unfolded protein” and “protein processing in ER” pathways are listed in Supplementary Table S4. Further analysis revealed that apoptosis-related genes were also upregulated, while the downregulated signaling pathways included the response to ER stress, the ER-nucleus signaling pathway, the ubiquitin-dependent ERAD (ER-associated degradation) pathway, regulation of the ER unfolded protein response, and protein folding in the ER (Figure S5c-d).

Finally, with respect to simultaneously inhibiting or silencing LSD1 and G9a, our analysis revealed a total of 981 upregulated genes and 795 downregulated genes (Figure 4(g)). GSEA revealed that genes involved in “apoptotic signaling pathway,” “response to ER stress,” and “negative regulation of cell cycle” were differentially regulated (Figure 4(h)), including *SFRP2*, *TNF*, and *DUSP1* (Figure 4(i)). The *SFRP2* (secreted frizzled-related protein 2) gene encodes an antagonist in the Wnt signaling pathway and has been reported to play an antitumorigenic role in colorectal cancer; moreover, its expression is associated with increased H3K4me2 levels at the gene's promoter, which is occupied by LSD1 in specific regions [26]. Recently, Liu et al. reported a loss of SFRP2 expression in esophageal squamous cell carcinoma tissues [27]. These findings are supported by our RNA-seq analysis showing that inhibiting both LSD1 and G9a causes the significant upregulation of SFRP2 expression. Together, these data indicate that SFRP2 may play a protective role against the progression of esophageal cancer. Notably, genes involved in apoptosis and in the negative regulation of cell proliferation were also significantly upregulated upon inhibition of both LSD1 and G9a (Figure S5e); in contrast, genes involved in the ubiquitin-dependent ERAD pathway were significantly downregulated (Figure S5f).

### 2.6. Inhibiting Both LSD1 and G9a in ESCC Cells Induces S-Phase Arrest and Apoptosis

**Figure 5:**
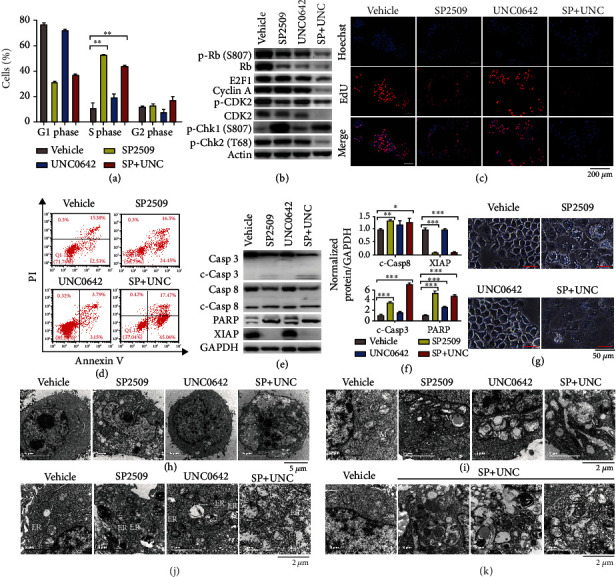
Inhibiting both LSD1 and G9a in ESCC cells induces S-phase arrest and apoptosis. (a) Summary of the percentage of ESCC cells in the G1, S, or G2 phase after the indicated treatments for 2 days. SP2509 5 *μ*M, UNC0642 5 *μ*M. (b) Western blot analysis of the indicated cell cycle-associated proteins in ESCC cells treated as indicated. (c) EdU staining of ESCC cells treated with vehicle, 3 *μ*M SP2509, 1.2 *μ*M UNC0642, or both for 2 days; the nuclei were counterstained with Hoechst 33342. (d) Apoptosis analysis of ESCC cells after the indicated treatments for 2 days. (e) Western blot analysis of the indicated apoptosis-associated proteins in ESCC cells treated as indicated for 2 days. (f) Quantification of the indicated proteins measured in (e), expressed relative to vehicle-treated cells. (g) Representative images of ESCC cells treated with vehicle, 10 *μ*M SP2509, 10 *μ*M UNC0642, or both for 2 days. (h–k) Representative transmission electron microscopy images of ESCC cells treated as in (g); mitochondria (M) and endoplasmic reticulum (ER) are indicated. The scale bars are 5 *μ*m in (h), 2 *μ*m in (i–k). ^∗^*p* < 0.05, ^∗∗^*p* < 0.01, and ^∗∗∗^*p* < 0.001 (unpaired Student's *t*-test).

To identify the molecular mechanism by which inhibiting LSD1 and G9a in ESCC cells causes cell death, we examined cell cycle progression, cell proliferation, and cellular apoptosis using flow cytometry. We found that treating cells with both SP2509 and UNC0642 caused a marked increase in S-phase arrest compared to vehicle-treated control cells (Figure 5(a)); moreover, treating cells with either SP2509 alone or UNC0642 alone induced S-phase arrest in a dose-dependent manner, with UNC0642 having a less potent effect than SP2509 (Figure S6a-b). To confirm these findings, we measured S-phase-associated proteins and found that p-CDK2 was downregulated, and p-Chk1 was upregulated in cells treated with SP2509 alone and in cells treated with both SP2509 and UNC0642. Moreover, cells treated with both SP2509 and UNC0642 had reduced levels of all S-phase-associated proteins measured, including total Rb, p-Rb, E2F1, cyclin A, CDK2, p-CDK2, and p-Chk2 (Figure 5(b)). In addition, we performed an EdU incorporation assay and found that proliferation was reduced in cells treated with SP2509 alone and in cells treated with both SP2509 and UNC0642 (Figure 5(c)). Finally, the DNA synthesis inhibitor hydroxyurea further increased cell death induced by SP2509 and/or UNC0642 (Figure S6c).

Using flow cytometry, we also found that cotreating ESCC cells with SP2509 and UNC0642 significantly increased apoptosis (Figure 5(d) and Figure S6d); moreover, both SP2509 alone and UNC0642 alone induced apoptosis in a dose-dependent manner, with UNC0642 having a more potent effect than SP2509 (Figure S6e). In addition, various apoptosis inhibitors, including Z-VAD-FMK, Z-DEVD-FMK, and emricasan, significantly reduced apoptosis induced by UNC0642, but had no effect on apoptosis induced by treating cells with SP2509 alone or treating cells with both SP2509 and UNC0642 (Figure S6f-g). We then measured apoptosis-associated proteins in ESCC cells and found that cotreating cells with SP2509 and UNC0642 group increased cleaved caspase-3, cleaved caspase-8, and cleaved PARP protein levels and decreased XIAP protein levels (Figures 5(e) and 5(f)), supporting the notion that simultaneously inhibiting LSD1 and G9a induces apoptosis in ESCC cells. As an additional test of this hypothesis, we performed a viability assay using LCL161, a SMAC (second mitochondrial activator of caspase) mimetic that binds to and inhibits IAPs (inhibitor of apoptosis proteins) such as XIAP and c-IAP. We found that LCL161 significantly increased the death of ESCC cells induced by SP2509 and/or UNC0642 (Figure S6h).

Interestingly, we found that treating cultured ESCC cells with SP2509 led to reduced cell numbers and enlarged cell size, with increased numbers of vacuoles; in contrast, treating cells with UNC0642 had little effect on cellular morphology, while cotreating cells with both SP2509 and UNC0642 led to massive levels of cell death and severe morphological changes (Figure 5(g)). We examined these changes in further detail using transmission electron microscopy and found abnormal cellular morphology in cells treated for 2 days with SP2509 alone, UNC0642 alone, or both SP2509 and UNC0642, with the most severe changes observed in the cells treated with both inhibitors; in addition, we observed nuclear pyknosis in all treated groups, as well as vacuoles in cells treated with SP2509 either alone or in combination with UNC0642 (Figure 5(h)). We also observed severely damaged mitochondria in cells cotreated with SP2509 and UNC0642, including disrupted cristae, mitochondrial swelling, and mitochondrial shrinkage (Figure 5(i)). Finally, we observed swollen ER (Figure 5(j)) and severely damaged organelles in cells cotreated with SP2509 and UNC0642 (Figure 5(k)).

Taken together, these results indicate that inhibiting both LSD1 and G9a causes death in ESCC cells by inducing apoptosis, S-phase arrest, and severe damage to multiple organelles.

### 2.7. Targeting ER Stress Sensitizes ESCC Cells to the Effect of Inhibiting LSD1 and G9a

**Figure 6:**
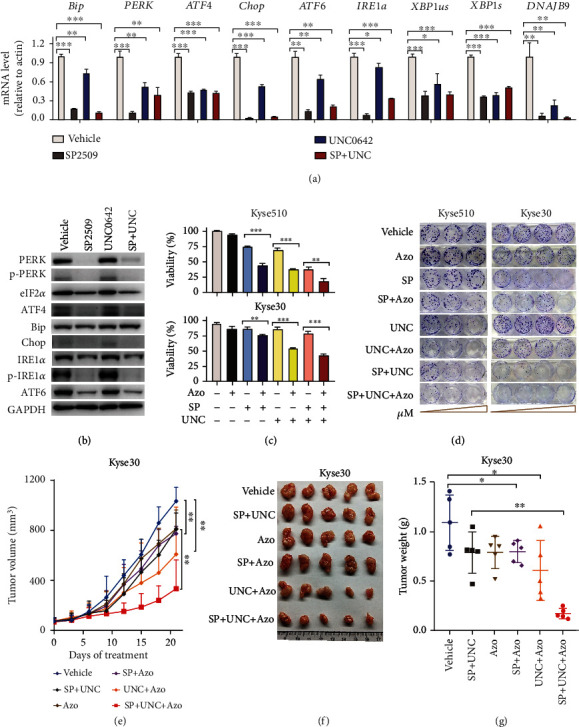
Targeting modulators of ER stress sensitizes ESCC cells to LSD1 and G9a inhibitors. (a) mRNA levels of the indicated ER stress-associated genes were measured using qRT-PCR in ESCC cells treated with 10 *μ*M SP2509, 10 *μ*M UNC0642, or both SP2509 and UNC0642 for 3 days. (b) Western blot analysis of the indicated proteins measured in ESCC cells treated as indicated for 3 days. (c) Summary of the viability of Kyse510 and Kyse30 cells treated as indicated for 3 days; Kyse510 cells were treated with 6 *μ*M azoramide (Azo), 0.9 *μ*M SP2509, and/or 0.6 *μ*M UNC0642; Kyse30 cells were treated with 16 *μ*M Azo, 0.8 *μ*M SP2509, and/or 0.8 *μ*M UNC0642. (d) Colony formation assay of ESCC cells treated with increasing concentrations of the indicated compounds. (e–g) Mice received a xenograft of Kyse30 cells and were subsequently treated with vehicle or the indicated combinations of Azo (60 mg/kg daily), SP2509 (25 mg/kg twice weekly), and/or UNC0642 (2.5 mg/kg, every 2 days); tumor volume (e), photographed tumors (f), and final tumor weight (g) were measured (*n* = 5 per group). ^∗^*p* < 0.05, ^∗∗^*p* < 0.01, and ^∗∗∗^*p* < 0.001 (unpaired Student's *t*-test).

Lastly, we examined whether ER stress plays a role in mediating the effects of inhibiting LSD1 and G9a in ESCC cells by measuring the mRNA levels of various ER stress-related genes in cells treated with vehicle, SP2509, UNC0642, or both inhibitors. Consistent with our RNA-seq data, we found that treated cells had significantly downregulated mRNA levels of the ER stress-related genes, including*Bip*, *PERK*, *ATF4*, *Chop*, *ATF6*, *IRE1α*, unspliced *XBP1* (*XBP1us*), spliced *XBP1* (*XBP1s*), and *DNAJB9* (Figure 6(a)). In addition, Western blot analysis showed that PERK, p-PERK, eIF2*α*, ATF4, Chop, IER1*α*, p-IER1*α*, and ATF6 protein levels were decreased in cells treated with SP2509 alone and in cells treated with both SP2509 and UNC0642; in contrast, treating cells with UNC0642 alone had no effect on the levels of these ER stress-related proteins (Figure 6b). Given that “response to unfolded protein” and “protein processing in ER” ranked at the top of the pathways significantly affected by inhibiting G9a, we examined the effect of treating cells with AUY-922, an inhibitor of heat shock protein 90 (HSP90), a chaperone protein that helps stabilize partially folded proteins. We found that AUY-922 significantly increased UNC0642-induced cell death (Figure S7a) and suppressed colony formation in a concentration-dependent manner (Figure S7b), indicating that the unfolded protein response is affected by inhibiting G9a in ESCC cells.

Next, we tested whether inducing ER stress altered the effects of inhibiting LSD1 and/or G9a in ESCC cells. We found that treating cells with tunicamycin—an inducer of ER stress—decreased cell viability but had no further effect on cell viability in cells treated with SP2509 alone, UNC0642 alone, or both SP2509 and UNC0642 (Figure S7c). However, we found that although treating ESCC cells with GSK2606414—a cell-permeable inhibitor of PERK (protein kinase R-like ER kinase), a sensor of ER stress—also had no effect on cell viability, it increased the effects of SP2509 and UNC0642 with respect to reducing cell viability (Figure S7d). In addition, we found that the ER stress modulator azoramide (Azo)—which improves ER protein folding and activates ER chaperones to protect cells against ER stress in multiple systems—not only induced cell death in a concentration-dependent manner (Figure S7e) but also significantly increased the sensitivity of ESCC cells to SP2509 and/or UNC0642 (Figure 6(c) and Figure S7f). Moreover, treating cells with Azo together with SP2509 and/or UNC0642 significantly reduced colony formation (Figure 6(d) and Figure S7g). Given that the Bcl-2 protein family has been reported to play an important role in controlling ER stress-induced apoptosis [28], we also measured the protein levels of Bcl-2 and Bax, but found no significant difference between groups (Figure S7h).

Finally, we examined whether modulating ER stress *in vivo* can affect tumor growth in our xenograft mouse model. We found that treating xenograft recipient mice with Azo together with SP2509 alone, UNC0642 alone, or both SP2509 and UNC0642 significantly reduced Kyse30-derived tumor growth compared to control-treated mice, with the strongest effect observed in mice that received triple treatment with Azo, SP2509, and UNC0642 (Figures 6(e)–6(g)), with no effect on body weight (Figure S8g); similar results were obtained in mice carrying tumors derived from Kyse180 cells (Figure S8). Taken together, these results support the notion that inhibiting LSD1 and G9a sensitizes ESCC cells to the effects of inhibiting ER stress both *in vitro* and *in vivo*, indicating that ER stress plays a role in ESCC.

## 3. Discussion

Here, we report that LSD1 and G9a synergistically promote cell growth in ESCC, and we provide preclinical evidence supporting the combined inhibition of LSD1 and G9a as a novel and promising strategy for treating ESCC. In addition, we show that ESCC cells require LSD1 and G9a for survival based on shRNA-mediated gene silencing. With respect to the underlying mechanism, we show that coinhibiting LSD1 and G9a induces apoptosis and S-phase arrest and downregulates ER stress-related pathways.

LSD1, the first identified histone demethylase, is well characterized as a transcriptional repressor by interacting with either the CoREST or NuRD repressor complex and by demethylating enhancer-associated H3K4me1/H3K4me2 [29–31]. Moreover, LSD1 may also function as an upstream activator of androgen receptor signaling in prostate cancer [32, 33]. Interestingly, a growing body of evidence suggests that LSD1 can serve as an oncogene by demethylating nonhistone proteins such as p53, DNMT1, E2F1, STAT3, and MYPT1 in a variety of cancer types [34–36]. However, although LSD1 has been linked to esophageal cancer, the underlying mechanisms and clinical implications are unknown [37, 38]. Here, we provide functional data showing that LSD1 is required for ESCC growth, based on our *in vitro* and *in vivo* experiments using the LSD1-specific inhibitor SP2509 and shRNA-mediated LSD1 knockdown, indicating that LSD1 may serve as a viable target and prognostic biomarker for ESCC.

G9a was the second identified histone lysine methyltransferase, and its primary function is the methylation of H3K9, a marker of silent euchromatin, which is important for transcription, signal transduction, cell proliferation, and differentiation [39]. Similar to LSD1, G9a can also catalyze nonhistone substrates, including p53, WIZ, CDYL1, CSB, ACINUS, HDAC1, DNMT1, and KLF12 [40]. High expression levels of G9a have been reported in a variety of cancer types, including colon cancer, liver cancer, and metastatic ovarian cancer [41–43]. In addition to its function as a repressor of specific transcription factors, G9a can also serve as an activator, requiring other coactivators such as CARM1 and P300 to drive gene expression [44, 45]. Although Zhong et al. previously reported that G9a is associated with ESCC, its role in tumorigenesis is currently unclear [46]. Using the G9a-specific inhibitor UNC0642 and shRNA to inhibition and knockdown G9a, respectively, we found that targeting G9a promotes ESCC cell death by inducing apoptosis.

Importantly, we also found that cotreatment with both SP2509 and UNC0642 induced apoptosis and S-phase arrest in ESCC cells both *in vitro* and in our *in vivo* xenograft model. In addition, we found that severe cell morphological changes were present in ESCCs co-treated with both SP2509 and UNC0642, including increased vacuoles and nuclear pyknosis, mitochondrial changes, and swollen ER.

Our RNA-seq analysis revealed that ER stress-related signaling pathways were markedly changed in ESCC cells treated with SP2509 and/or UNC0642, and these pathways ranked as the most downregulated pathways in cells treated with either drug alone. The ER stress signaling pathways—collectively known as the unfolded protein response (UPR)—are required for maintaining ER function [47]; if ER function is disrupted, the UPR pathway triggers cell death. The ER stress-induced cellular response is mediated by three ER transmembrane receptors called PERK, ATF6, and IRE1 [48]. Moreover, ATF4 was previously shown to be upregulated in ESCC, contributing to the progression of esophageal cancer [49]. However, whether the ER stress pathway plays a functional role in the pathogenesis of esophageal cancer remains an open question. Here, we found that genes involved in the ER stress and UPR pathways, including *PERK*, *ATF4*, *IRE1α*, and *ATF6*, are significantly downregulated in ESCC cells treated with LSD1 and/or G9a inhibitors. Notably, we also found that modulators of ER stress act synergistically with LSD1 and G9a inhibitors to further increase the death of ESCC cells both *in vitro* and *in vivo*, indicating that modulating ER stress may serve as a promising therapeutic strategy for ESCC when combined with inhibitors of LSD1 and/or G9a. On the other hand, although Bcl-2 protein family members have been reported to play an important role in controlling ER stress-induced apoptosis [28], our finding of no significant change in Bcl-2 or Bax protein levels indicates that apoptosis induced by ER stress might not be play a role in the death of ESCC cells treated with either the LSD1 inhibitor alone or both the LSD1 and G9a inhibitors; thus, the precise underlying mechanism remains unknown.

With respect to the clinical significance, we found that both the mRNA and protein levels of LSD1 and G9a are upregulated in esophageal cancer samples, and their expression levels were correlated with each other. In addition, we analyzed clinical data obtained from 114 ESCC samples and found that LSD1 expression was significantly correlated with patient age and the tumor's T stage. Recently, Sheng et al. reported that either knocking out or pharmacologically inhibiting LSD1 in a mouse model of melanoma overcame the tumor's resistance to anti-PD-1 therapy by increasing tumor immunogenicity and T cell infiltration, suggesting that combining LSD1 inhibition with PD-1 and/or PD-L1 blockers might serve as an effective strategy for treating certain types of cancer [50]. We therefore tested whether LSD1 expression is correlated with either PD-L1 or CD8 expression, but found no such correlation. With respect to G9a, we found that this enzyme's expression is correlated with tumor grade and Ki67—but not with PD-L1 or CD8—in patient samples. Recently, Segovia et al. found that inhibiting G9a and the methyltransferase DNMT improved the efficacy of PD-1-based immunotherapy in treating bladder cancer [51]. Moreover, Kelly et al. recently reported that inhibiting G9a increases the antitumor activity of anti-PD-1 by modulating autophagy and interferon signaling in melanoma [52]. With respect to esophageal cancer, both the phase 1b KEYNOTE-028 study and the phase 2 KEYNOTE-180 study suggest that pembrolizumab (a monoclonal antibody against PD-1) can produce a durable response in both advanced and metastatic esophageal carcinoma [6, 53]. The recent ESCORT-1st study (ClinicalTrials.gov identifier: NCT03691090) found that adding camrelizumab (another monoclonal antibody against PD-1) to the first-line chemotherapy (paclitaxel and cisplatin) significantly improved overall survival and progress-free survival compared to chemotherapy alone in patients with advanced and/or metastatic ESCC [54]. In addition, a multicenter phase 3 trial (JUPITER-06) demonstrated the efficacy and safety of toripalimab plus paclitaxel/cisplatin as the first-line treatment for patients with advanced ESCC; compared with paclitaxel/cisplatin alone, toripalimab plus paclitaxel/cisplatin extended progression-free survival and overall survival, irrespective of PD-L1 expression [55]. Given our finding that high expression of both LSD1 and G9a was correlated with the lowest survival in patients with ESCC, future clinical studies are clearly needed in order to determine whether targeting both LSD1 and G9a can improve PD-1-based therapies.

In conclusion, we show that inhibiting both LSD1 and G9a significantly reduces the growth of ESCC both *in vitro* and *in vivo*. Although the precise mechanisms by which these two enzymes affect ESCC require further study, our findings are clinically relevant. Moreover, our finding that high expression of both LSD1 and G9a is associated with poor outcome in patients with ESCC suggests that inhibiting both LSD1 and G9a may represent a promising strategy for treating these patients.

## 4. Materials and Methods

### 4.1. Cell Experiments

All cell lines (AGS, A549, Bxpc-3, EL-4, HCT116, Huh-7, HepG2, HCC-7703, HCC-7404, HT1080, HT-29, HGC, Jurkat, Kyse30, Kyse150, Kyse180, Kyse510, MDA-MB-231, LM3, LM6, Molt-4, MKN-45, SK-MES-1, TE-13, and 769-P) were purchased from the Cell Bank at the Chinese Academy of Sciences (Shanghai, China) and cultured in the recommended culture media contained 10% (*v*/*v*) fetal bovine serum at 37°C in 5% CO_2_.

For measuring cell viability, ESCC cells (1000-2000 cells/well) were seeded in 96-well plates. The following day, the cells were treated with the indicated compounds at the indicated concentrations for the indicated times, and cell viability was measured using the MTT assay (Sangon Biotech, Shanghai, China) in accordance with the manufacturer's instructions.

### 4.2. Antibodies and Reagents

The following antibodies were used for immunostaining: anti-Ki67 (BD eBioscience, Franklin Lakes, NJ), anti-H3K4me2 (HuaBio Biotech, Hangzhou, China), anti-H3K9me2 (Abcam, Cambridge, UK), anti-LSD1 (Cell Signaling Technology, Beverly, MA), and anti-G9a (Abcam).

The following antibodies were used for Western blot analysis: anti-LSD1 (Cell Signaling Technology), anti-G9a (Abcam), anti-H3K9me2 (Immunoway, Plano, TX), anti-H3K4me2 (Immunoway), anti-pRb (Immunoway), anti-Rb (HuaAn Biotech, Hangzhou, China), anti-E2F1 (HuaAn Biotech), anti-Cyclin A (Santa Cruz, Dallas, TX), anti-p-CDK2 (Cell Signaling Technology), anti-CDK2 (Santa Cruz), anti-Caspase-3 (Cell Signaling Technology), anti-Caspase-8 (Proteintech, Rosemont, IL), anti-XIAP (Bioworld, Bloomington, MN), anti-PARP (Cell Signaling Technology), anti-PERK (Santa Cruz), anti-p-PERK (Affinity Biosciences, Cincinnati, OH), anti-eIF2a (HuaAn Biotech), anti-ATF4 (Cell Signaling Technology), anti-Bip (HuaAn Biotech), anti-Chop (Santa Cruz), anti-IRE1a (Santa Cruz), anti-p-IRE1a (Novus, Centennial, CO), anti-ATF6 (HuaAn Biotech), and anti-GAPDH (Proteintech), followed by the appropriate HRP-conjugated secondary antibodies (Beyotime Biotech, Shanghai, China).

The following reagents were also used: hematoxylin and eosin (H&E) staining kit (Beyotime Biotech), TdT in situ apoptosis detection kit (R&D Systems, Minneapolis, MN), TRIzol reagent (Pufei Biotech, Shanghai, China), SYBR Green Supermix (Bimake, Houston, TX), RIPA buffer (Beyotime Biotech), protease inhibitors (Bimake), BCA protein assay kit (Beyotime Biotech), polyvinylidene fluoride (PVDF) membranes (Bio-Rad, Hercules, CA), Pierce ECL System (Thermo Scientific, Waltham, MA), EdU Kit (Ruibo Biotechnology Co., Ltd., Guangzhou, China), cell cycle staining kit (Multi Sciences Biotech, Hangzhou, China), annexin V-FITC/PI apoptosis detection kit (Multi Sciences Biotech), and PrimeScript RT reagent kit (Takara, Kyoto, Japan). All other chemicals and compounds used in this study are listed in Supplementary Table S1.

### 4.3. Colony Formation Assay

Cells (300-900 cells/well) were incubated in 12-well or 6-well plates at 37°C for 1 day and then treated with either vehicle or the indicated combinations of SP2509, BIX01294, UNC0642, azoramide, and/or AUY-922 at the indicated concentrations. For cells expressing Dox-inducible shRNA constructs, the cells were treated with vehicle or 200 ng/ml doxycycline. For cells overexpressing LSD1 or G9a, the cells were transfected with an empty control vector, pEnter-LSD1 (CH887551, Vigene Biosciences, Rockville, MD), or pEGFP-hG9a (a gift from Martin Walsh; Addgene plasmid #33025; http://n2t.net/addgene:33025 [56]) for 2 days and then seeded at 900 cells/well. The culture medium was changed every 3 days. When the cells grew to the stage of visible colonies, the colonies were washed gently with phosphate-buffered saline (PBS) and fixed with prechilled methanol (-20°C) for 10 min at room temperature. The methanol was removed, and the cells were stained for 15 min at room temperature by the addition of 1 ml 0.5% crystal violet diluted in 20% methanol. After removing the staining solution, the colonies were washed, air-dried, and counted under a light microscope.

### 4.4. RNA Isolation and Quantitative RT-PCR

Total RNA was extracted using TRIzol reagent, and RNA concentration and purity were measured using spectrophotometry. RNA was reverse-transcribed using the PrimeScript RT Kit, and quantitative real-time was performed using a CFX96 real-time system (Bio-Rad) with SYBR Green Supermix in accordance with the manufacturer's instructions. The fold difference in gene expression was calculated using the 2^−△△Ct^ method and is presented relative to *GAPDH* mRNA. All reactions were performed in triplicate, and specificity was monitored using melting curve analysis. The qRT-PCR primers used in this study are listed in Table S5.

### 4.5. H&E Staining and Immunohistochemistry of Tumor Tissues

Paraffin-embedded tumors were sectioned at 4 *μ*M and stained with H&E. For immunohistochemistry, the tumor sections were placed on polylysine-coated slides, deparaffinized in xylene, rehydrated using a graded ethanol series, quenched in 3% hydrogen peroxide to eliminate endogenous peroxidase activity, and processed for antigen retrieval by microwaving for 7 min in 10 mM citrate buffer (pH 6.0). The sections were then stained with the following primary antibodies: anti-Ki67, anti-H3K4me2, or anti-H3K9me2. The appropriate HRP-conjugated anti-rabbit or anti-mouse secondary antibody was then used and visualized using the REAL EnVision Detection System (K5007, Dako, Santa Clara, CA); the sections were counterstained with hematoxylin. The TdT In Situ Apoptosis Detection Kit was also used to stain select tumor sections. All photomicrographs were obtained using an Eclipse E400 microscope (Nikon).

### 4.6. Western Blot Analysis

Total proteins were extracted by homogenizing the cells in RIPA buffer containing protease inhibitors. The homogenate was cleared by centrifugation at 12,000 rpm at 4°C for 20 min, and the supernatant (containing the protein fraction) was collected. Protein concentration in the supernatant was measured using the BCA Protein Assay Kit. Equal amounts (ranging from 30-100 *μ*g) of denatured proteins were loaded into each lane of a 10% SDS polyacrylamide gel, separated by electrophoresis, and transferred to PVDF membranes. The membranes were blocked with 5% (*w*/*v*) nonfat milk in Tris-buffered saline containing 0.1% Tween-20 and then incubated overnight at 4°C in primary antibodies, followed by 2 h at room temperature in secondary antibodies; the signal was detected using the Pierce ECL System.

### 4.7. EdU Incorporation Assay

EdU incorporation was measured using the EdU Kit (Ruibo Biotechnology Co., Ltd., Guangzhou, China) in accordance with the manufacturer's instructions. In brief, the cells (1000 cells/well) were seeded in 96-well plates, incubated at 37°C for 1 day, and then treated with vehicle, SP2509 (3 *μ*M), UNC0642 (1.2 *μ*M), or both SP2509 and UNC0642 for 2 days. The cells were then labeled with EdU at 37°C for 2 h and fixed for 20 min in 4% paraformaldehyde (PFA), followed by 5 min in glycine. After washing, the cells were stained with Hoechst 33342 for 30 min in the dark. Photomicrographs were obtained using an Axio Observer A1 fluorescence microscope (Zeiss), and the percentage of EdU-positive cells was calculated.

### 4.8. Cell Cycle Analysis

Cell cycle was analyzed using the Cell Cycle Staining Kit (Multi Sciences Biotech, Hangzhou, China) in accordance with the manufacturer's instructions. In brief, cells were treated with the indicated inhibitors for 2 days, collected, washed with PBS, labeled with 1 ml DNA staining solution containing 10 *μ*l permeabilization solution for 30 min in the dark at room temperature, and analyzed using flow cytometry (CytoFLEX LX FACS, Beckman Coulter).

### 4.9. Annexin V-FITC Apoptosis Staining

Apoptosis was measured using an Annexin V-FITC/PI apoptosis detection kit (Multi Sciences Biotech). After 2 days of treatment, ESCC cells were harvested by trypsinization without EDTA and washed twice with ice-cold PBS. The cells were then prepared as a single-cell suspension and dual-stained with 5 *μ*l Annexin V-FITC and 10 *μ*l propidium iodide (PI) in the dark for 5 min at room temperature. The percentage of apoptotic cells was determined using flow cytometry (CytoFLEX LX FACS).

### 4.10. Transmission Electron Microscopy

Cells were treated with the indicated inhibitors for 2 days, collected, fixed in a solution containing 2.5% glutaraldehyde in PBS overnight at 4°C, and then, washed 3 times (10 min/wash) in 1 ml 0.1 M PBS. The cells were postfixed with 1% osmium tetroxide for 1 h, washed 3 times with distilled water, stained with 2% uranyl acetate, dehydrated using a 50%–100% ethyl alcohol gradient and 100% acetone, and then, infiltrated and embedded. The embedded samples were trimmed and sectioned on a Leica UC7 ultramicrotome. Thin sections were obtained and collected on a 200-mesh nickel grid. The grids were contrasted with 5% uranyl acetate for 20 mins and lead citrate for 6 mins. Finally, the samples were visualized using a Tecnai G2 Spirit transmission electron microscope (Thermo FEI).

### 4.11. Lentivirus-Mediated shRNA for Gene Silencing

HEK293T cells were transfected with the lentiviral Tet-pLKO-puro vector containing the relevant shRNA hairpin sequence together with the psPAX2 packaging plasmid (TV00426, TranSheep Bio, Shanghai, China) and the pMD2.G envelope plasmid (TV00772, TranSheep Bio). The supernatant containing the lentivirus was collected, concentrated, and used to infect esophageal cancer cells. Stably infected cells were screened using puromycin (ST551, Beyotime Biotech). The shRNA sequences used to knock down *LSD1* and *G9a* are listed in Supplementary Table S6.

### 4.12. ESCC Cell Xenograft Mouse Models

Female 5-week-old BALB/c nude mice were purchased from Vital River Laboratory Animal Technology Co., Ltd. (Beijing, China) and housed under specific pathogen-free conditions at the Laboratory Animal Center of Zhejiang University. All animal experiments were performed in accordance with the National Institutes of Health's Guide for the Care and Use of Laboratory Animals and were approved by the Ethics Committee of the Laboratory Animal Center of Zhejiang University. For cell injections, 5 × 10^6^, 4 × 10^6^, or 1 × 10^7^ Kyse510, Kyse30, or Kyse180 cells, respectively, were suspended in 100 *μ*l PBS, mixed with 50% Matrigel (#354248, BD Biosciences), and injected subcutaneously into the mouse's flank. Tumor size (length × width^2^ × 0.5) was measured using a digital vernier caliper. When the tumors reached approximately 150 mm^3^, the mice were randomly assigned to the control (vehicle) and experiment groups (5 mice per group), which then received intraperitoneal injections of vehicle (solvent) or various combinations of SP2509 (25 mg/kg, twice weekly), UNC0642 (2.5 mg/kg, every 2 days), and/or azoramide (60 mg/kg, daily). For mice injected with shRNA-expressing ESCC cells, the mice in the control group were given normal drinking water, while the mice in the experiment group received water containing 2 mg/ml doxycycline (Dox). The health status of each animal, including any change in body weight, was monitored daily. When the tumors reached approximately 1500 mm^3^, the mice were euthanized, and the tumors were harvested, photographed, fixed in 4% PFA, and embedded in paraffin for subsequent immunohistochemistry.

### 4.13. Tissue Microarray Analysis

A tissue microarray containing human esophageal cancer tissues (HEsoS180Su08), including 66 pairs of esophageal cancer tissues with matched adjacent healthy tissues, as well as additional 48 esophageal cancer tissues, was obtained from Shanghai Outdo Biotech (Shanghai, China). The microarray project was reviewed and approved by the Clinical Research Ethics Committee and Institutional Review Board of Shanghai Outdo Biotech. All patients gave written informed consent. The Dako ChemMate EnVision Detection Kit with peroxidase/diaminobenzidine (DAB) was used in accordance with the manufacturer's instructions. Immunostained tissue microarrays were assessed by two pathologists who were blinded with respect to the clinical information and scored by multiplying the intensity (range: 0–3) and extent (range: 0–100) of staining for each tissue.

### 4.14. RNA Sequencing and Bioinformatics Analysis

Total RNA was extracted and sequenced using the Illumina platform (Anoroad Genome Technologies Corporation, Beijing, China). Raw RNA-seq reads were then processed by removing low-quality reads and joint contamination, yielding high-quality (clean) reads, which were then used for subsequent analyses. These high-quality reads were mapped to the *Homo sapiens* (GRCh38.87.chr) genome. Differential expression was determined, and the fold change was calculated relative to the respective control conditions. Genes were considered to be significantly upregulated or downregulated at *p* < 0.05 and with a fold change (in either direction) of ≥1.3 relative to the corresponding control. Gene Set Enrichment Analysis was performed using Metascape (https://metascape.org). The RNA-seq data (GSE173666) are available via Gene Expression Omnibus.

### 4.15. Statistical Analyses

All data were analyzed and plotted using SPSS or GraphPad Prism, and all summary data are presented as the mean ± standard deviation. To meet the assumption of homogeneity of variance, an analysis of variance (ANOVA) was performed, followed by Tukey's multiple comparison test. An unpaired Student's *t*-test was used to compare the differences between two groups. Pearson's test was used to analyze potential correlations, and the pair-wise log-rank test was used to analyze patient survival. Differences were considered statistically significant at *p* < 0.05.

## Figures and Tables

**Table 1:** Correlation analysis between LSD1 or G9a expression and clinical pathological features of ESCC patients.

	LSD1					G9a				
	LSD1 expression		Total	*χ* ^2^	*p* value	G9a expression		Total	*χ* ^2^	*p* value
	Low	High				Low	High			
Age (years)				3.959	**0.047**				2.114	0.146
≤65	17	40	57			27	30	57		
>65	8	48	56			19	37	56		
Sex				0.534	0.465				2.851	0.091
Female	8	22	30			16	14	30		
Male	17	67	84			30	54	84		
Grade				0.021	0.884				4.811	**0.028**
1-2	20	70	90			41	49	90		
3-4	5	19	24			5	19	24		
T stage				4.196	**0.041**				0.456	0.499
T1-T2	9	14	23			11	12	23		
T3-T4	16	69	85			34	51	85		
N stage				1.164	0.281				0.382	0.537
N0	14	39	53			23	30	53		
N1-N3	11	50	61			23	38	61		
TNM stage				2.713	0.100				1.109	0.292
1-2	16	38	54			25	29	54		
3-4	9	46	55			20	35	55		
p53				2.085	0.149				0.474	0.491
Negative	8	16	24			9	15	24		
Positive	11	48	59			27	32	59		
Ki67				0.013	0.908				5.597	**0.018**
Negative	8	26	34			20	14	34		
Positive	11	38	49			16	33	49		
PD-L1				1.516	0.218				0.024	0.878
Negative	15	41	56			23	33	56		
Positive	10	48	58			23	35	58		
CD8				0.010	0.920				0.180	0.671
Negative	20	72	92			38	54	92		
Positive	5	17	22			8	14	22		

## Data Availability

The RNA-seq data used in this study have been deposited in Gene Expression Omnibus and are available via GSE173666 (https://www.ncbi.nlm.nih.gov/geo/query/acc.cgi?acc=GSE173666). The flow cytometry data to explore cell cycle and apoptosis of this study have been deposited in FlowRepository (Repository ID: FR-FCM-Z3PN, URL: https://flowrepository.org/id/FR-FCM-Z3PN). Additional data are related to this paper. The datasets used and/or analyzed in this study are available within the manuscript and its supplementary information files. Correspondence and requests for materials should be addressed to J.M. (junxiamin@zju.edu.cn).

## Supplementary Materials

Supplementary Materials Figure S1: inhibiting both LSD1 and G9a significantly reduces the viability of esophageal cancer cells. Figure S2: genetically silencing LSD1 and G9a increases ESCCs cell death. Figure S3: systemic treatment with doxycycline or inhibitors of LSD1 and/or G9a does not affect the body weight of mice bearing tumors derived from xenografted esophageal cancer cells. Figure S4: LSD1 and G9a expression is upregulated in esophageal cancer tissues obtained from The Cancer Genome Atlas database. Figure S5: Gene Set Enrichment Analysis of RNA-seq data in ESCCs following knockdown and pharmacological inhibition of LSD1 alone, G9a alone, or both LSD1 and G9a. Figure S6: targeting LSD1 and/or G9a induces S-phase arrest and apoptosis in ESCCs. Figure S7: inhibitors of ER-stress sensitize esophageal cancer cells to pharmacological inhibitors of LSD1 and G9a. Figure S8: targeting modulators of ER-stress modulators sensitizes ESCCs to the effects of inhibiting LSD1 and G9a *in vivo*. Table S1: list of the chemicals and compounds used in this study. Table S2: clinicopathological information of all 114 patients with esophageal squamous cell carcinoma. Table S3: univariate and multivariate analyses of the factors correlated with overall survival of ESCC patients. Table S4: list of genes that were significantly upregulated or downregulated (defined as a fold change in either direction ≥ 1.3 and *p* < 0.05) in ESCCs in response to genetic silencing and inhibition of G9a. Table S5: list of the primer pairs used for quantitative real-time PCR analysis. Table S6: list of target-specific shRNA hairpin sequences for knocking down human LSD1 (hLSD1) and G9a (hG9a).
