# Supplementary material for: Targeting the LSD1-G9a-ER Stress Pathway as a Novel Therapeutic Strategy for Esophageal Squamous Cell Carcinoma
Source: Research (Wash D C). 2022 Jun 1;2022:9814652. doi: 10.34133/2022/9814652 (PMC9185438; doi:10.34133/2022/9814652)
Supplement: Supplementary Materials — Figure S1: inhibiting both LSD1 and G9a significantly reduces the viability of esophageal cancer cells. Figure S2: genetically silencing LSD1 and G9a increases ESCCs cell death. Figure S3: systemic treatment with doxycycline or inhibitors of LSD1 and/or G9a does not affect the body weight of mice bearing tumors derived from xenografted esophageal cancer cells. Figure S4: LSD1 and G9a expression is upregulated in esophageal cancer tissues obtained from The Cancer Genome Atlas database. Figure S5: Gene Set Enrichment Analysis of RNA-seq data in ESCCs following knockdown and pharmacological inhibition of LSD1 alone, G9a alone, or both LSD1 and G9a. Figure S6: targeting LSD1 and/or G9a induces S-phase arrest and apoptosis in ESCCs. Figure S7: inhibitors of ER-stress sensitize esophageal cancer cells to pharmacological inhibitors of LSD1 and G9a. Figure S8: targeting modulators of ER-stress modulators sensitizes ESCCs to the effects of inhibiting LSD1 and G9a in vivo. Table S1: list of the chemicals and compounds used in this study. Table S2: clinicopathological information of all 114 patients with esophageal squamous cell carcinoma. Table S3: univariate and multivariate analyses of the factors correlated with overall survival of ESCC patients. Table S4: list of genes that were significantly upregulated or downregulated (defined as a fold change in either direction ≥ 1.3 and p < 0.05) in ESCCs in response to genetic silencing and inhibition of G9a. Table S5: list of the primer pairs used for quantitative real-time PCR analysis. Table S6: list of target-specific shRNA hairpin sequences for knocking down human LSD1 (hLSD1) and G9a (hG9a). [file 9814652.f1.zip › Wang et al Supplementary Files-NEW.docx]

**Supplementary Materials for**

**Targeting the LSD1-G9a-ER Stress Pathway as a Novel Therapeutic Strategy for Esophageal Squamous Cell Carcinoma**

Hongxiao Wang, Zijun Song, Enjun Xie, Junyi Chen, Biyao Tang, Fudi Wang, Junxia Min

**This file includes:**

Figures S1 to S8

Tables S1 to S6

**Supplementary Fig. S1**

**
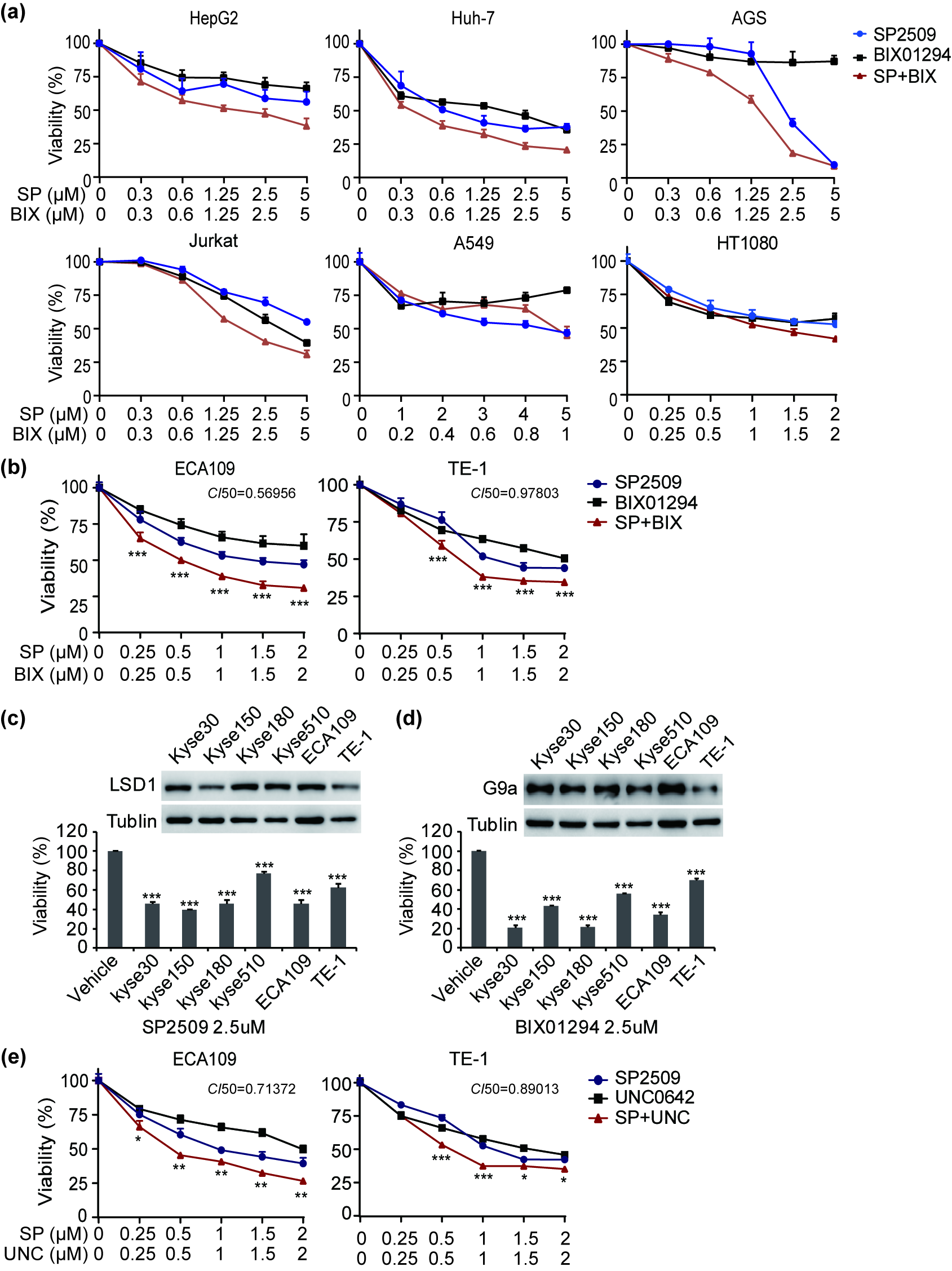
**

**Supplementary Figure S1. Inhibiting both LSD1 and G9a significantly reduces the viability of esophageal cancer cells**.

**(a)** Cell viability of HepG2 and Huh-7 (hepatoma cell lines), AGS (a gastric cancer cell line), Jurkat (a lymphoma cell line), A549 (a lung cancer cell line), and HT1080 (a fibroblast carcinoma cell line) cells treated for 3 days with the indicated concentrations of SP2509 and/or BIX01294 (n=3/group). **(b)** Cell viability of the esophageal squamous carcinoma cell lines ECA109 and TE-1 treated with SP2509 and/or BIX01294 at the indicated concentrations for 3 days (n=3/group). **(c-d)** LSD1 (c) and G9a (d) protein levels and cell viability were measured in the indicated esophageal cancer cells. For the viability assay, the indicated cell lines were treated with 2.5 μM SP2509 or 2.5 μM BIX01294 for 3 days. **(e)** Cell viability of ECA109 and TE-1 cells treated with SP2509 and/or UNCO642 at the indicated concentrations for 3 days (n=3/group). *CI*50: combination index at median effect of drug treatment. *CI*50<1 indicates synergism **p*<0.05, ***p*<0.01, and ****p*<0.001 (unpaired Student’s *t*-test).

**Supplementary Fig. S2
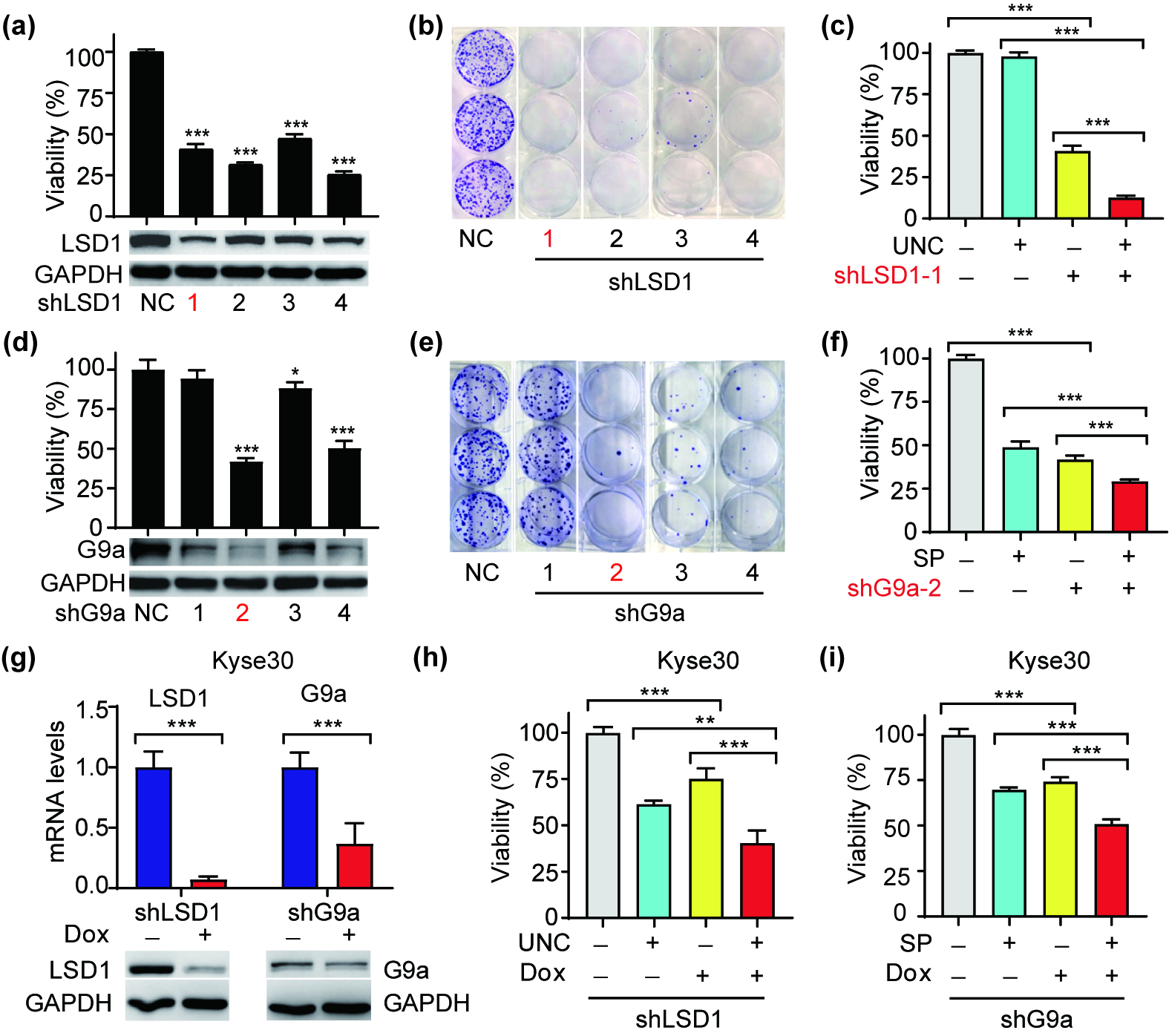
**

**Supplementary Figure S2. Genetically silencing LSD1 and G9a increases ESCCs cell death.**

**(a)** ESCCs were infected with a virus expressing a non-silencing control shRNA (NC) or four different shLSD1 shRNA constructs, and cell viability and LSD1 protein levels were measured and normalized to control cells. **(b)** Colony formation assay of ESCCs expressing the indicated shRNAs. **(c)** Summary of the viability of ESCCs infected with the shLSD1-1 shRNA and then treated with vehicle or UNC0642 for 3 days. **(d-f)** Same as (a-c), except the cells were infected with shG9a shRNA constructs. **(g)** Silencing efficiency of LSD1 and G9a in Kyse30 cells was determined by measuring mRNA and protein levels after treatment with vehicle or 200 ng/ml doxycycline (Dox) for 3 days. **(h-i)** Viability of Kyse30 cells stably expressing Dox-inducible shLSD1 (h) or shG9a (i) where indicated, cells were treated for 3 days with 200 ng/ml Dox, 3 μM UNC0642, or 1 μM SP2509. ***p*<0.01 and ****p*<0.001 (unpaired Student’s *t*-test).

**Supplementary Fig. S3**


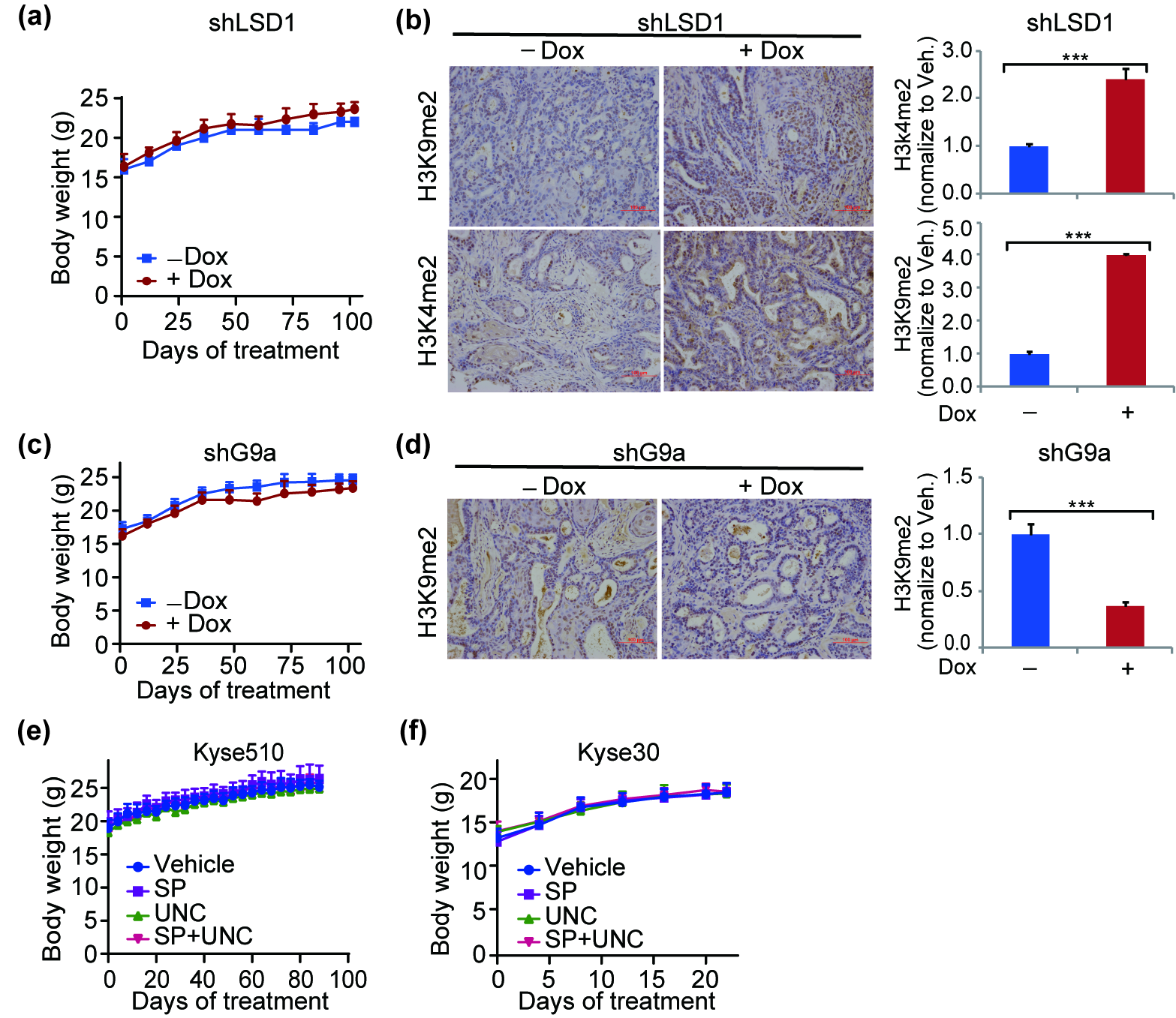


**Supplementary Figure S3. Systemic treatment with doxycycline or inhibitors of LSD1 and/or G9a does not affect the body weight of mice bearing tumors derived from xenografted esophageal cancer cells.**

**(a)** Body weight of mice injected with ESCCs stably expressing the Dox-inducible shLSD1 construct, and then treated with either vehicle (-Dox) or doxycycline (+Dox). **(b)** Example IHC images (left) and summary (right) of H3K4me2 and H3K9me2 proteins measured in tumors taken from the mice in (a). **(c)** Body weight of mice injected with ESCCs stably expressing the Dox-inducible shG9a construct, and then treated with either vehicle (-Dox) or doxycycline (+Dox). **(d)** Example IHC images (left) and summary (right) of H3K9me2 protein measured in tumors taken from the mice in (c). **(e-f)** Body weight of mice injected with either Kyse510 (e) or Kyse30 (f) ESCCs and then treated as indicated. ****p*<0.001 (unpaired Student’s *t*-test).

**Supplementary Fig. S4**


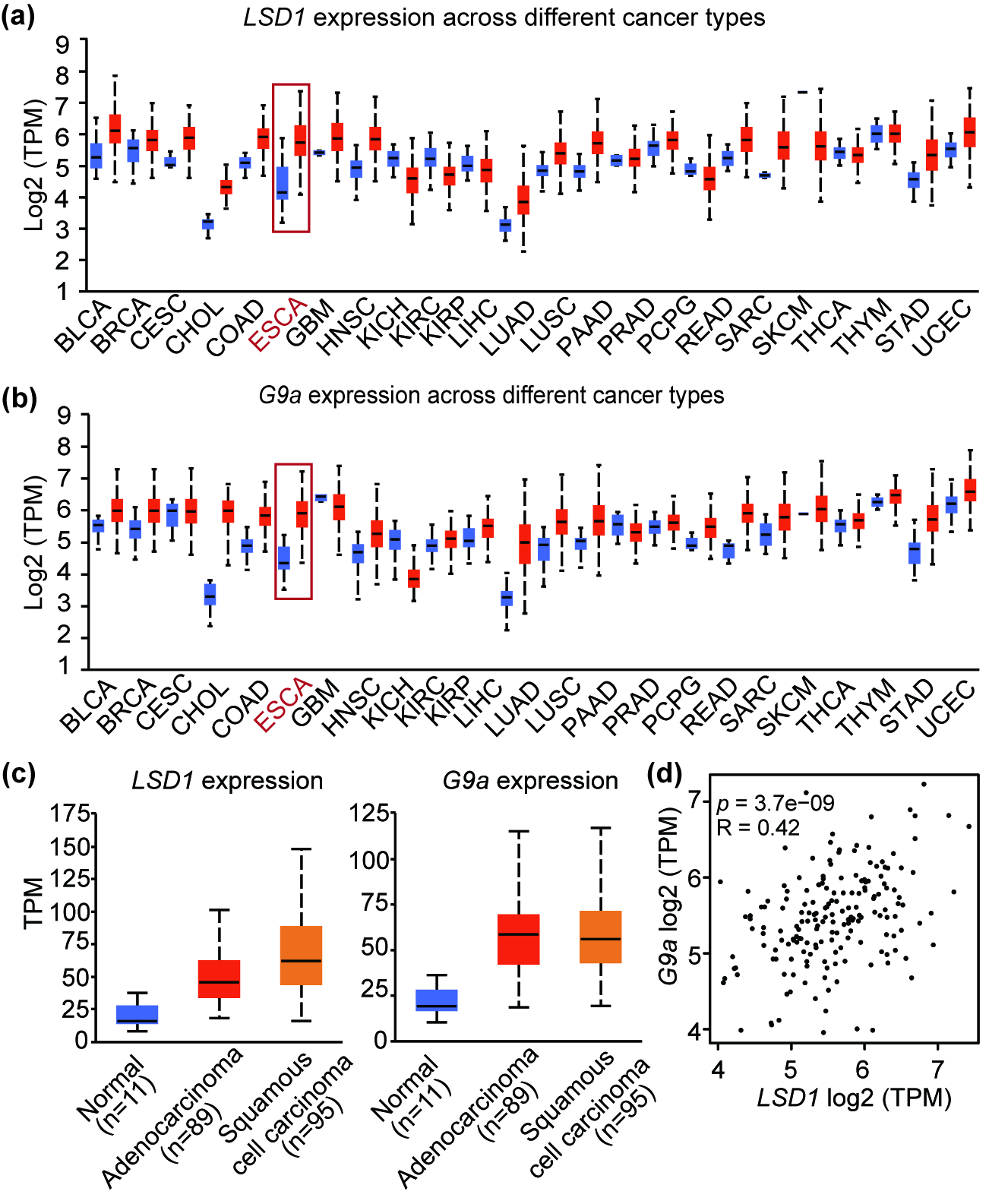


**Supplementary Figure S4. LSD1 and G9a expression is upregulated in esophageal cancer tissues obtained from The Cancer Genome Atlas database.**

**(a-b)** Summary of *LSD1* (a) and *G9a* (b) mRNA levels measured in the indicated tumor tissues (red) and corresponding healthy tissues (blue) obtained from patients with the indicated cancer types. The esophageal squamous cancer and adenocarcinoma (ESCA) samples and matched control samples are indicated with a red box. **(c)** Summary of *LSD1* (left) and *G9a* (right) mRNA measured in healthy tissues, adenocarcinoma tissues, and squamous cell carcinoma tissues; data are presented as transcripts per million (TPM). **(d)** Correlation analysis between *LSD1* mRNA and *G9a* mRNA levels in esophageal cancer tissues retrieved from the TCGA dataset, with the corresponding Pearson’s correlation coefficient.

**Supplementary Fig. S5**

**
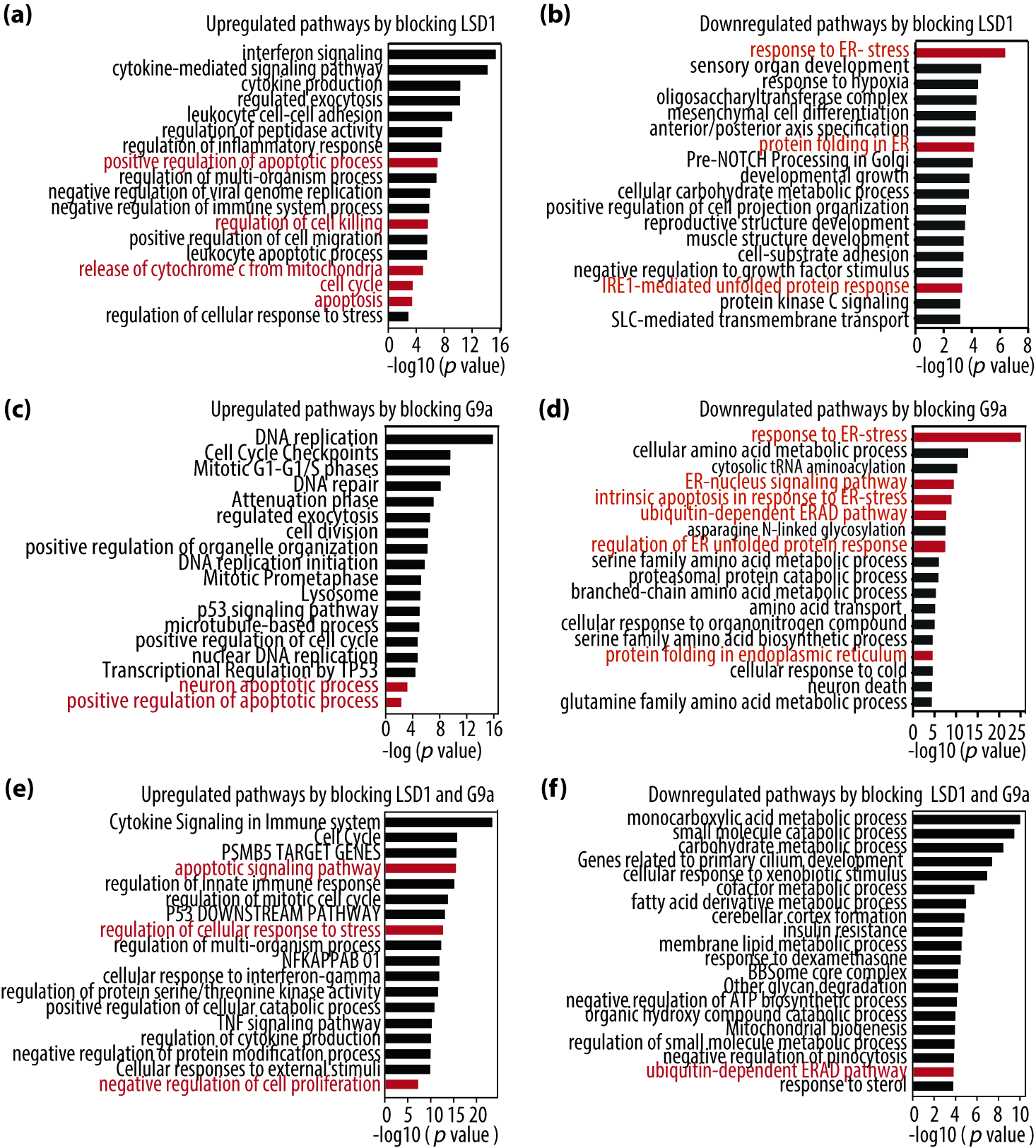
**

**Supplementary Figure S5. Gene Set Enrichment Analysis of RNA-seq data in ESCCs following knockdown and pharmacological inhibition of LSD1 alone, G9a alone, or both LSD1 and G9a.**

**(a-b)** Gene Set Enrichment Analysis of upregulated (a) and downregulated (b) transcripts in ESCCs in which LSD1 was blocked using either shLSD1 shRNA or SP2509 treatment. **(c-d)** Gene Set Enrichment Analysis of upregulated (c) and downregulated (d) transcripts in ESCCs in which G9a was blocked using either shG9a shRNA or UNC0642 treatment. **(e-f)** Gene Set Enrichment Analysis of upregulated (e) and downregulated (f) transcripts in ESCCs in which both LSD1 and G9a were blocked using either shLSD1+shG9a shRNA or SP2509+UNC0642 treatment. In all cases, downregulation and upregulation were determined relative to the corresponding control group.

**Supplementary Fig. S6**


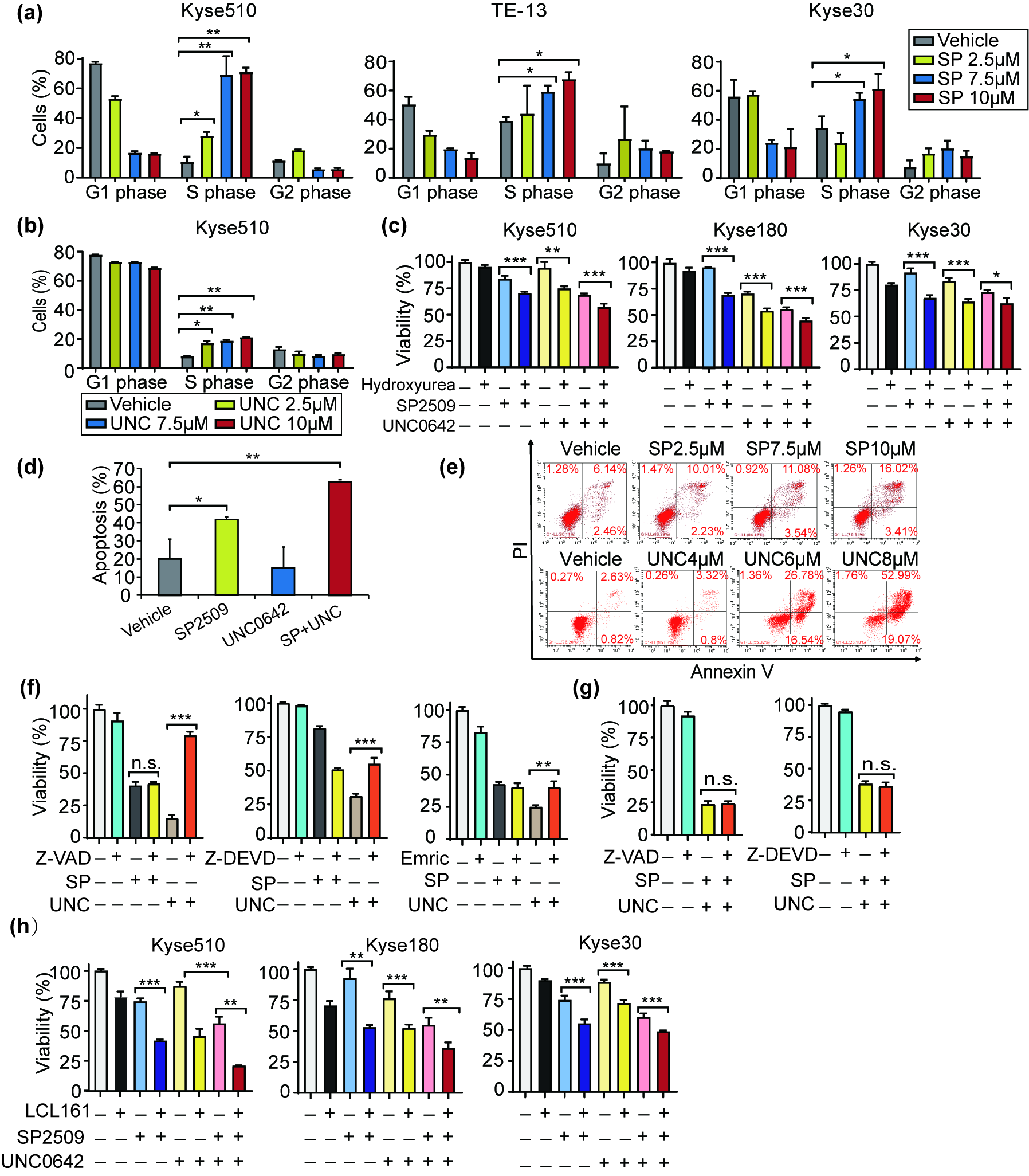


**Supplementary Figure S6. Targeting LSD1 and/or G9a induces S-phase arrest and apoptosis in ESCCs.**

**(a-b)** Summary of the percentage of ESCCs in the G1, S, or G2 phase after the indicated treatment for 2 days (n=3/group). **(c)** Summary of the viability of the indicated cell lines treated with SP2509, UNC0642, and/or the DNA synthesis inhibitor hydroxyurea for 3 days (n=3/group). **(d)** Quantification of apoptosis was shown relative to vehicle-treated cells in ESCCs after the indicated treatments for 2 days. SP2509 5μM, UNC0642 5μM .**(e)** Apoptosis analysis of ESCCs treated with the indicated concentrations of SP2509 or UNC0642 for 2 days. **(f)** Summary of the viability of ESCCs treated with SP2509 or UNC0642 for 3 days in the absence or presence of the apoptosis inhibitors Z-VAD-FMK (Z-VAD, left), Z-DEVD-FMK (Z-DEVD, middle), and emricasan (Emric, right). **(g)** Cell viability of ESCCs treated with both SP2509 and UNC0642 as indicated for 3 days in the absence or presence of the apoptosis inhibitors Z-VAD-FMK (left), Z-DEVD-FMK (right). **(h)** Summary of the viability of the indicated cell lines treated with SP2509, UNC0642, and/or LCL161 for 3 days. Kyse510 (LCL161 30μΜ, SP 1.8μΜ，UNC 1.8μΜ), Kyse180 (LCL161 10μΜ, SP 0.6μΜ，UNC 0.6μΜ), Kyse30 (LCL161 20μΜ, SP 1.2μΜ，UNC 1.2μΜ) SP**p*<0.05, ***p*<0.01, and ****p*<0.001 (unpaired Student’s *t*-test).

**Supplementary Fig. S7**

**
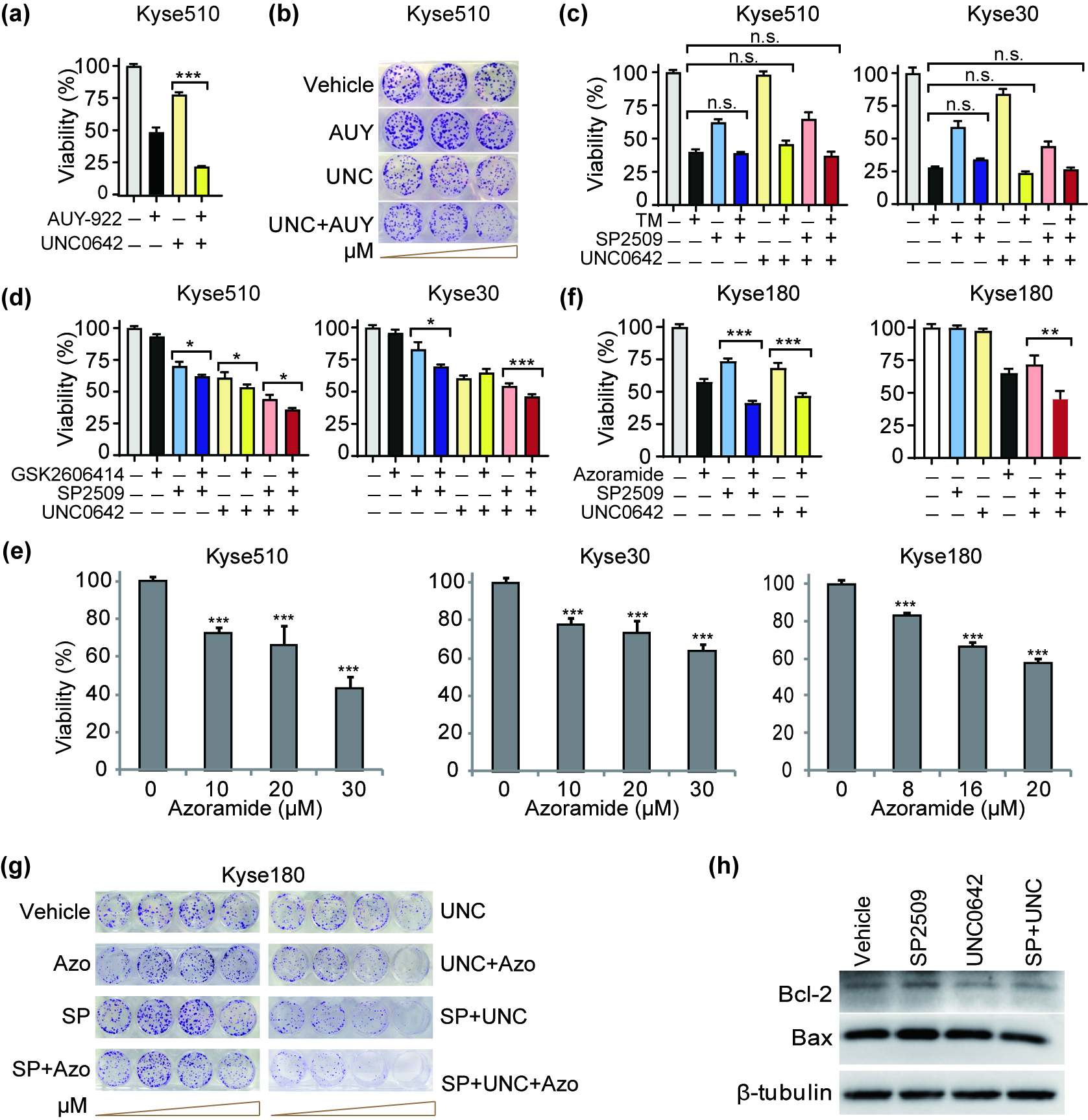
**

**Supplementary Figure S7. Inhibitors of ER-stress sensitize esophageal cancer cells to pharmacological inhibitors of LSD1 and G9a.**

**(a)** Summary of the viability of Kyse510 cells treated with UNC0642 and/or AUY-922 for 3 days. **(b)** Colony formation assay of Kyse510 cells treated with vehicle or increasing concentrations of UNC0642 and/or AUY-922（AUY） for 10 days. **(c-d)** Summary of the viability of the indicated ESCCs treated with SP2509 and/or UNC0642 in the absence or presence of tunicamycin (TM; c), GSK2606414 (d). **(e)** Azoramide’s effect on ESCCs. **(f)** Summary of the viability of the indicated ESCCs treated with SP2509 and/or UNC0642 with or without Azoramide for 3 days. **(g)** Colony formation assay of Kyse180 cells treated with vehicle or increasing concentrations of SP2509, UNC0642, and/or azoramide (Azo) for 10 days. **(h)** Western blot analysis of Bcl-2 and Bax in ESCCs treated as indicated. The data represent three independent experiments. **p*<0.05, ***p*< 0.01, ****p*<0.001, and n.s., not significant (unpaired Student’s t-test).

**Supplementary Fig. S8**


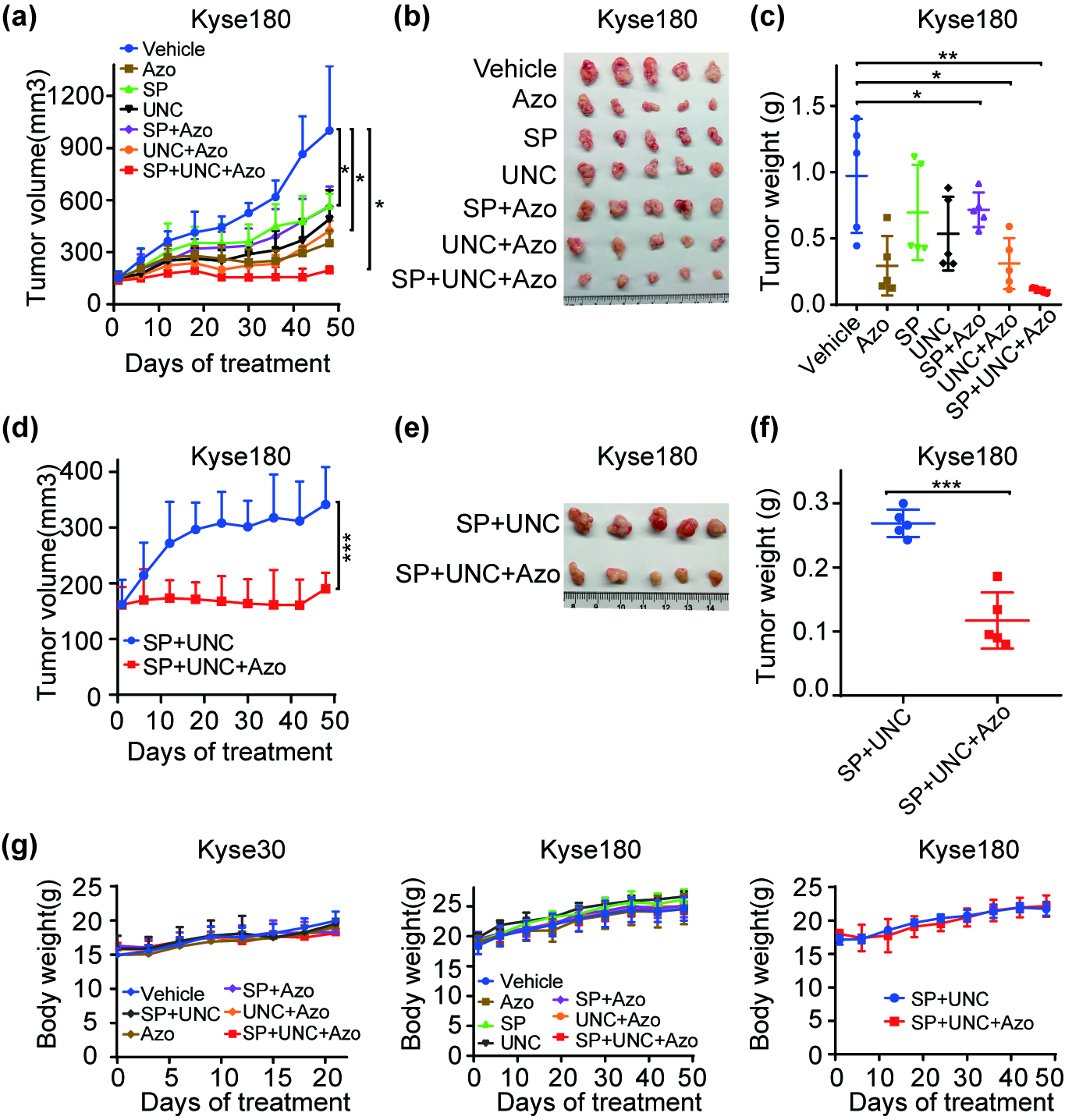


**Supplementary Figure S8. Targeting modulators of ER-stress modulators sensitizes ESCCs to the effects of inhibiting LSD1 and G9a *in vivo***

**(a-f)** Mice received an injection of Kyse180 ESCCs and were subsequently treated with vehicle or the indicated combinations of Azo (60 mg/kg daily), SP2509 (25 mg/kg twice weekly), and UNC0642 (2.5 mg/kg, every 2 days). Tumor volume ((a) and (d)), photographed tumors ((b) and (e)), and final tumor weight ((c) and (f)) were measured (n=5 per group). **(g)** Body weight of mice bearing tumors derived from the indicated ESCC lines and treated with the indicated compounds. **p*<0.05, ***p*< 0.01, and ****p*<0.001 (unpaired Student’s t-test).

**Table S1. List of the chemicals and compounds used in this study**

| **Name** | **M.W.** | **Cat. No.** | **CAS No.** | **Activity** | **Source** |
| --- | --- | --- | --- | --- | --- |
| BML-210 | 339.4 | GR-330 | N.A. | HDAC inhibitor | National Compound Resource Center, Shanghai, China |
| Suberoyl bis Hydroxamic acid | 204.2 | GR-323 | 38937-66-5 | HDAC inhibitor | National Compound Resource Center, Shanghai, China |
| Scriptaid | 326.4 | GR-326 | N.A. | HDAC inhibitor | National Compound Resource Center |
| Phenylbutyrate Na | 186.2 | EI-320 | 1716-12-7 | HDAC inhibitor | National Compound Resource Center, Shanghai, China |
| Valproic acid | 144.2 | GR-352 | 99-66-1 | HDAC inhibitor | National Compound Resource Center, Shanghai, China |
| MC-1293 | 284.3 | 270-344 | N.A. | HDAC inhibitor | National Compound Resource Center, Shanghai, China |
| NSC-3852 | 174.2 | AC-1391 | 3565-26-2 | HDAC inhibitor | National Compound Resource Center, Shanghai, China |
| Valproxam | 159.2 | GR-351 | N.A. | HDAC inhibitor | National Compound Resource Center, Shanghai, China |
| NCH-51 | 390.6 | AC-1389 | 848354-66-5 | HDAC inhibitor | National Compound Resource Center, Shanghai, China |
| Nullscript | 298.3 | GR-327 | N.A. | Analog of scriptaid lacks of HDAC inhibitory effects of scriptaid | National Compound Resource Center, Shanghai, China |
| ITSA1 | 292.1 | GR-350 | 200626-61-5 | Inhibitor of TSA activity | National Compound Resource Center |
| Nicotinamide | 122.1 | KI-283 | 98-92-0 | SIRT inhibitor | National Compound Resource Center, Shanghai, China |
| BML-266 | 478.5 | GR-346 | N.A. | SIRT2 inhibitor | National Compound Resource Center, Shanghai, China |
| AGK2 | 434.3 | 270-484 | 304896-28-4 | SIRT2 inhibitor | National Compound Resource Center |
| Suramin Na | 1429.2 | G-430 | 129-46-4 | SIRT1 inhibitor | National Compound Resource Center, Shanghai, China |
| Splitomycin | 198.2 | GR-331 | N.A. | SIRT-2 inhibitor | National Compound Resource Center |
| EX-527 | 248.7 | GR-348 | N.A. | SIRT1 inhibitor | National Compound Resource Center, Shanghai, China |
| Sirtinol | 394.5 | 270-308 | 410536-97-9 | SIRT inhibitor | National Compound Resource Center |
| Salermide | 394.5 | AC-1388 | 1105698-15-4 | SIRT inhibitor | National Compound Resource Center, Shanghai, China |
| Iso-Nicotinamide | 122.12 | AC-1392 | 1453-82-3 | Nicotinamide antagonist | National Compound Resource Center |
| B2 | 396.8 | 270-485 | 115687-05-3 | SIRT2 inhibitor | National Compound Resource Center |
| Resveratrol | 228.2 | FR-104 | 501-36-0 | SIRT1 activator | National Compound Resource Center, Shanghai, China |
| Piceatannol | 244.3 | EI-271 | 10083-24-6 | SIRT activator | National Compound Resource Center |
| Aminoresveratrol | 227.2 | GR-363 | N.A. | SIRT1 activator | National Compound Resource Center, Shanghai, China |
| BML-278 | 391.5 | GR-359 | N.A. | SIRT1 activator | National Compound Resource Center, Shanghai, China |
| CTPB | 554.1 | 420-033 | 586976-24-1 | HAT inhibitor | National Compound Resource Center, Shanghai, China |
| Anacardic acid | 348.5 | 270-381 | N.A. | HAT inhibitor | National Compound Resource Center, Shanghai, China |
| Garcinol | 602.8 | GR-343 | 78824-30-3 | HAT inhibitor | National Compound Resource Center, Shanghai, China |
| BRD4770 | 413.47 | S7591 | 1374601-40-7 | Histone methyltransferase G9a inhibitor | Selleck, Houston, USA |
| BIX01294 | 490.64 | S8006 | 935693-62-2 | Histone methyltransferase G9a inhibitor | Selleck, Houston, USA |
| 2,4-Pyridinedicarboxylic Acid | 167.1 | A-280 | 499-80-9 | Histone demethylase inhibitor | National Compound Resource Center, Shanghai, China |
| ORY-1001 | 303.27 | S7795 | 1431303-72-8 | Lysine demethylase LSD1 inhibitor | Selleck, Houston, USA |
| Tranylcypromine | 169.7 | EI-217 | 13492-01-8 | Lysine demethylase inhibitor | National Compound Resource Center, Shanghai, China |
| GSK2879552 | 413.47 | S7591 | 1401966-69-5 | Histone methyltransferase G9a inhibitor | Selleck, Houston, USA |
| SP2509 | 437.9 | HY-12635 | 1423715-09-6 | Lysine demethylase LSD1 inhibitor | MedChemExpress, Monmouth Junction, USA |
| 5-Aza-2-deoxycytidine | 228.2 | GR-345 | 2353-33-5 | DNA methyltransferase inhibitor | National Compound Resource Center, Shanghai, China |
| Zebularine | 228.2 | GR-344 | 3690-10-6 | DNA methyltransferase inhibitor | National Compound Resource Center, Shanghai, China |
| FK866 | 391.51 | S2799 | 658084-64-1 | NMPRTase inhibitor | Selleck, Houston, USA |
| P7C3 | 474.19 | S7968 | 301353-96-8 | NMPRTase inhibitor | Selleck, Houston, USA |
| Azoramide | 308.83 | HY-18705 | 932986-18-0 | Modulator of UPR | MedChemExpress, Monmouth Junction, USA |
| Z-VAD-FMK | 467.49 | S7023 | 187389-52-2 | Irreversible Pan-caspase inhibitor | Selleck, Houston, USA |
| Z-DEVD-FMK | 668.66 | T6005 | 210344-95-9 | Caspase-3 inhibitor | Topscience, Shanghai, China |
| Emricasan | 569.50 | S7775 | 254750-02-2 | Irreversible Pan-caspase inhibitor | Selleck, Houston, USA |
| AUY-922 | 465.54 | S1069 | 747412-49-3 | HSP90 inhibitor | Selleck, Houston, USA |

M.W., molecular weight; Cat. No., catalog number; CAS No., Chemical Abstracts Service number; HAT, histone acetyltransferase; HDAC, histone deacetylase; HSP90, heat shock protein-90; N.A., not applicable; NMPRTase, nicotinamide phosphoribosyltransferase; SIRT, sirtuin (NAD-dependent protein deacetylase); TSA, trichostatin A.

**Table S2. Clinicopathological information of all 114 patients with esophageal squamous cell carcinoma.**

| Case number | Survival time (months) | Status | Gender | Age | Tumor | Lymph  node | Metastasis | Pathological grade |
| --- | --- | --- | --- | --- | --- | --- | --- | --- |
| 1 | 19 | D | M | 60 | T3 | N1 | M0 | Ⅱ |
| 2 | 58 | D | M | 70 | T3 | N0 | M0 | Ⅱ-Ⅲ |
| 3 | 26 | D | M | 73 | T3 | N1 | M0 | Ⅱ-Ⅲ |
| 4 | 13 | D | F | 74 | T3 | N.A. | M0 | Ⅰ-Ⅱ |
| 5 | 107 | S | M | 66 | T3 | N0 | M0 | Ⅰ-Ⅱ |
| 6 | 15 | D | M | 73 | T3 | N0 | M0 | Ⅰ-Ⅱ |
| 7 | 60 | D | M | 78 | N.A. | N1 | M0 | Ⅱ |
| 8 | 36 | D | M | 67 | T2 | N0 | M0 | Ⅰ-Ⅱ |
| 9 | 18 | D | M | N.A. | T3 | N1 | M0 | Ⅰ-Ⅲ |
| 10 | 5 | D | M | 72 | T3 | N1 | M0 | Ⅰ-Ⅱ |
| 11 | 1 | D | M | 74 | N.A. | N0 | M0 | Ⅱ |
| 12 | 104 | S | F | 68 | N.A. | N1 | M0 | Ⅰ-Ⅱ |
| 13 | 4 | D | M | 73 | T2 | N0 | M0 | Ⅰ-Ⅱ |
| 14 | 11 | D | M | 29 | T3 | N1 | M0 | Ⅰ-Ⅱ |
| 15 | 2 | D | M | 66 | T3 | N1 | M0 | Ⅰ-Ⅱ |
| 16 | 18 | D | M | 83 | T4 | N0 | M0 | Ⅱ |
| 17 | 4 | D | M | 65 | T3 | N0 | M0 | Ⅱ |
| 18 | 31 | D | M | 75 | T3 | N0 | M0 | Ⅱ |
| 19 | 51 | D | M | 51 | T3 | N0 | M0 | Ⅱ |
| 20 | 29 | D | M | 73 | N.A. | N0 | M0 | Ⅰ |
| 21 | 22 | D | M | 63 | T3 | N1 | M0 | Ⅱ |
| 22 | 35 | D | M | 51 | T3 | N2 | M0 | Ⅱ |
| 23 | 6 | D | M | 64 | T3 | N3 | M0 | Ⅱ-Ⅲ |
| 24 | 43 | D | M | 64 | T3 | N0 | M0 | Ⅱ |
| 25 | 44 | D | M | 55 | T2 | N0 | M0 | Ⅱ |
| 26 | 8 | D | M | 52 | T3 | N1 | M0 | Ⅱ |
| 27 | 9 | D | M | 59 | T3 | N3 | M0 | Ⅰ-Ⅱ |
| 28 | 16 | D | M | 70 | T3 | N1 | M0 | Ⅱ |
| 29 | 26 | D | Me | 63 | T2 | N0 | M0 | Ⅲ |
| 30 | 10 | D | F | 67 | T3 | N0 | M0 | Ⅱ-Ⅲ |
| 31 | 44 | D | M | 53 | T3 | N1 | M0 | Ⅰ |
| 32 | 95 | S | F | 71 | T3 | N0 | M0 | Ⅰ-Ⅱ |
| 33 | 23 | D | M | 69 | T3 | N1 | M0 | Ⅱ |
| 34 | 4 | D | M | 77 | T3 | N0 | M0 | Ⅰ-Ⅱ |
| 35 | 7 | D | F | 55 | T3 | N2 | M0 | Ⅰ |
| 36 | 31 | D | M | 64 | T3 | N1 | M0 | Ⅱ |
| 37 | 13 | D | M | 53 | T3 | N1 | M0 | Ⅱ |
| 38 | 50 | D | M | 66 | T2 | N0 | M0 | Ⅰ-Ⅱ |
| 39 | 15 | D | F | 56 | T3 | N0 | M0 | Ⅱ |
| 40 | 13 | D | F | 68 | T3 | N0 | M0 | Ⅰ-Ⅱ |
| 41 | 2 | D | M | 77 | T3 | N0 | M0 | Ⅰ-Ⅱ |
| 42 | 88 | S | M | 57 | T2 | N1 | M0 | Ⅰ-Ⅱ |
| 43 | 33 | D | M | 61 | T3 | N0 | M0 | Ⅰ-Ⅱ |
| 44 | 12 | D | M | 68 | T3 | N3 | M0 | Ⅲ |
| 45 | 12 | D | F | 72 | T3 | N0 | M0 | Ⅱ |
| 46 | 4 | D | M | 74 | T3 | N0 | M0 | Ⅱ |
| 47 | 5 | D | M | 72 | T2 | N2 | M0 | Ⅱ |
| 48 | 30 | D | F | 65 | T3 | N0 | M0 | Ⅰ-Ⅱ |
| 49 | 87 | S | F | 58 | T3 | N0 | M0 | Ⅱ |
| 50 | 22 | D | F | 63 | T3 | N0 | M0 | Ⅱ |
| 51 | 0 | D | M | 76 | T3 | N0 | M0 | Ⅰ-Ⅱ |
| 52 | 87 | S | F | 57 | T3 | N1 | M0 | Ⅱ-Ⅲ |
| 53 | 5 | D | M | 61 | T3 | N1 | M0 | Ⅱ |
| 54 | 15 | D | M | 79 | T2 | N3 | M0 | Ⅱ |
| 55 | 5 | D | M | 72 | T3 | N1 | M0 | Ⅱ-Ⅲ |
| 56 | 16 | D | M | 54 | T3 | N1 | M0 | Ⅱ |
| 57 | 84 | S | M | 76 | T2 | N1 | M0 | Ⅰ-Ⅱ |
| 58 | 26 | D | M | 65 | T3 | N3 | M0 | Ⅲ |
| 59 | 5 | D | M | 64 | T3 | N1 | M0 | Ⅰ |
| 60 | 83 | S | F | 71 | T1 | N0 | M0 | Ⅰ-Ⅱ |
| 61 | 15 | D | M | 48 | T3 | N2 | M0 | Ⅱ |
| 62 | 55 | D | M | 76 | T1 | N0 | M0 | Ⅱ-Ⅲ |
| 63 | 82 | S | F | 74 | T1 | N0 | M0 | Ⅱ-Ⅲ |
| 64 | 81 | S | M | 63 | T1 | N0 | M0 | Ⅱ |
| 65 | 10 | D | M | 52 | T3 | N1 | M0 | Ⅰ-Ⅱ |
| 66 | 79 | S | M | 69 | T2 | N1 | M0 | Ⅱ-Ⅲ |
| 67 | 6 | D | M | 69 | T3 | N1 | M0 | Ⅱ |
| 68 | 8 | D | F | 79 | T3 | N2 | M0 | Ⅱ |
| 69 | 7 | D | M | 78 | T3 | N0 | M0 | Ⅱ-Ⅲ |
| 70 | 6 | D | M | 84 | T3 | N2 | M0 | Ⅰ-Ⅱ |
| 71 | 6 | D | M | 74 | N.A. | N2 | M0 | Ⅱ |
| 72 | 2 | D | M | 50 | N.A. | N0 | M0 | Ⅰ |
| 73 | 47 | D | M | 51 | T3 | N2 | M0 | Ⅱ-Ⅲ |
| 74 | 10 | D | M | 60 | T3 | N2 | M0 | Ⅰ-Ⅱ |
| 75 | 2 | D | M | 72 | T3 | N2 | M0 | Ⅱ |
| 76 | 17 | D | F | 58 | T3 | N2 | M0 | Ⅱ |
| 77 | 23 | D | M | 71 | T3 | N2 | M0 | Ⅰ-Ⅲ |
| 78 | 9 | D | M | 51 | T2 | N0 | M0 | Ⅰ-Ⅱ |
| 79 | 97 | S | F | 62 | T2 | N0 | M0 | Ⅱ |
| 80 | 97 | S | M | 65 | T3 | N0 | M0 | Ⅰ-Ⅲ |
| 81 | 97 | S | F | 56 | T3 | N0 | M0 | Ⅱ-Ⅲ |
| 82 | 33 | D | M | 73 | T3 | N0 | M0 | Ⅰ-Ⅱ |
| 83 | 6 | D | M | 51 | T3 | N0 | M0 | Ⅰ-Ⅱ |
| 84 | 8 | D | M | 49 | T3 | N2 | M0 | Ⅱ |
| 85 | 2 | D | F | 82 | T3 | N2 | M0 | Ⅱ-Ⅲ |
| 86 | 43 | D | M | 48 | T3 | N1 | M0 | Ⅰ-Ⅱ |
| 87 | 9 | D | M | 61 | T3 | N1 | M0 | Ⅱ |
| 88 | 51 | D | F | 57 | T2 | N0 | M0 | Ⅱ |
| 89 | 12 | D | M | 65 | T3 | N0 | M0 | Ⅰ-Ⅱ |
| 90 | 41 | D | F | 63 | T1 | N0 | M0 | Ⅱ |
| 91 | 13 | D | M | 59 | T3 | N0 | M0 | Ⅱ |
| 92 | 88 | S | M | 57 | T3 | N0 | M0 | Ⅰ |
| 93 | 15 | D | M | 65 | T3 | N1 | M0 | Ⅱ-Ⅲ |
| 94 | 87 | S | M | 75 | T3 | N0 | M0 | Ⅱ |
| 95 | 5 | D | M | 80 | T3 | N1 | M0 | Ⅰ-Ⅱ |
| 96 | 5 | D | F | 73 | T3 | N2 | M0 | Ⅱ |
| 97 | 86 | S | F | 79 | T3 | N0 | M0 | Ⅱ-Ⅲ |
| 98 | 6 | D | F | 71 | T3 | N0 | M0 | Ⅰ |
| 99 | 7 | D | M | 50 | T3 | N2 | M0 | Ⅰ |
| 100 | 14 | D | M | 54 | T2 | N2 | M0 | Ⅱ |
| 101 | 84 | S | F | 81 | T2 | N0 | M0 | Ⅰ-Ⅱ |
| 102 | 1 | D | M | 81 | T3 | N1 | M0 | Ⅲ |
| 103 | 12 | D | M | 59 | T3 | N2 | M0 | Ⅱ |
| 104 | 84 | S | F | 68 | T3 | N1 | M0 | Ⅱ |
| 105 | 83 | S | F | 74 | T3 | N0 | M0 | Ⅱ |
| 106 | 4 | D | M | 62 | T3 | N2 | M0 | Ⅰ-Ⅱ |
| 107 | 83 | S | M | 52 | T2 | N1 | M0 | Ⅰ-Ⅱ |
| 108 | 10 | D | F | 74 | T3 | N0 | M0 | Ⅱ |
| 109 | 25 | D | F | 68 | T3 | N1 | M0 | Ⅲ |
| 110 | 10 | D | M | 74 | T3 | N2 | M0 | Ⅱ |
| 111 | 27 | D | M | 79 | T3 | N1 | M0 | Ⅲ |
| 112 | 28 | D | M | 65 | T3 | N1 | M0 | Ⅱ |
| 113 | 70 | D | F | 69 | T2 | N0 | M0 | Ⅱ |
| 114 | 58 | D | M | 77 | T2 | N0 | M0 | Ⅰ-Ⅱ |

N.A., not available; D, deceased; S, survived; M, male; F, female; T, tumor status; N, lymph node status; M, metastasis.

**Table S3. Univariate and multivariate analyses of the factors correlated with overall survival of ESCC patients**

| **Variables** | **Univariate analysis** | | | | |  | **Multivariate analysis** | | | |
| --- | --- | --- | --- | --- | --- | --- | --- | --- | --- | --- |
|  | *p* value | HR | | 95%CI | |  | *p* value | HR | 95%CI | |
|  |  |  | inferior limit | | [upper](javascript:;) [limit](javascript:;) |  |  |  | inferior limit | [upper](javascript:;) [limit](javascript:;) |
| **LSD1&G9A**  **（LSD1-high/G9A-low；LSD1-low/G9A-high；LSD1-high/G9A-high）** | **0.01** | 1.424 | | 1.086 | 1.866 |  | **0.042** | 1.351 | 1.011 | 1.804 |
| **Age**  （≤66 vs  >66） | 0.252 | 1.295 | | 0.832 | 2.016 |  |  |  |  |  |
| **Sex**  （Female vs  male） | **0.011** | 2.13 | | 1.191 | 3.807 |  | 0.056 | 1.802 | 0.986 | 3.294 |
| **Grade stage**  （I/II vs  III/IV） | 0.257 | 0.733 | | 0.429 | 1.254 |  |  |  |  |  |
| **T stage**  （T1/T2 vs  T3/T4） | **0.016** | 2.207 | | 1.157 | 4.207 |  | 0.114 | 1.824 | 0.865 | 3.845 |
| **N stage**  (N0 vs  N1/N2/N3) | **0.032** | 1.636 | | 1.042 | 2.569 |  | 0.371 | 0.649 | 0.251 | 1.675 |
| **TNM stage**  （I/II vs  III/IV） | **<0.0001** | 2.706 | | 1.667 | 4.395 |  | **0.045** | 2.889 | 1.024 | 8.151 |

Statistically significant (*p* < 0.05)

**Table S4. List of genes that were significantly upregulated or downregulated (defined as a fold change in either direction ≥1.3 and *p*<0.05) in ESCCs in response to genetic silencing and inhibition of G9a**

| **Gene** | **ID** | **Log2 fold change**  **(shRNA)** | ***P*-value** | **Log2 fold change (inhibitor)** | ***P*-value** |
| --- | --- | --- | --- | --- | --- |
| **Upregulated** | | | | | |
| *EPHB1* | ENSG00000154928 | 4.103603597 | 0.003730409 | 3.508601072 | 0.002023785 |
| *TFEC* | ENSG00000105967 | 3.933678596 | 0.006635629 | 5.452017543 | 5.54E-12 |
| *HSPA1L* | ENSG00000204390 | 2.411725893 | 2.56E-06 | 1.393123854 | 0.010904346 |
| *CCNE1* | ENSG00000105173 | 2.184903699 | 3.33E-26 | 1.790014007 | 2.76E-14 |
| *HSPA8* | ENSG00000109971 | 2.067004665 | 0 | 1.347743591 | 0 |
| *TUSC3* | ENSG00000104723 | 2.021141437 | 0.0047645 | 1.486233259 | 0.00360937 |
| *HLA-DOB* | ENSG00000241106 | 1.907683387 | 1.44E-06 | 1.682630471 | 0.008263572 |
| *DNAJA1* | ENSG00000086061 | 1.807591869 | 0 | 1.046350567 | 7.49E-141 |
| *SCAMP5* | ENSG00000198794 | 1.754129175 | 1.91E-15 | 2.018728339 | 5.55E-17 |
| *E2F1* | ENSG00000101412 | 1.685460187 | 2.71E-43 | 0.489742044 | 5.91E-06 |
| *HSPH1* | ENSG00000120694 | 1.653178664 | 5.77E-188 | 1.009502757 | 3.48E-139 |
| *MICB* | ENSG00000204516 | 1.552115801 | 1.89E-32 | 1.196431943 | 1.73E-21 |
| *HSP90AA1* | ENSG00000080824 | 1.440871012 | 0 | 0.662302797 | 0 |
| *CCNE2* | ENSG00000175305 | 1.398346863 | 1.53E-05 | 0.750675859 | 0.001040175 |
| *TUBB4B* | ENSG00000188229 | 1.392348271 | 0 | 0.654244871 | 1.75E-151 |
| *APAF1* | ENSG00000120868 | 1.298859889 | 3.90E-40 | 0.739952797 | 2.14E-13 |
| *UCP3* | ENSG00000175564 | 1.270713583 | 0.000763102 | 2.030553775 | 0.000692045 |
| *PSMD1* | ENSG00000173692 | 1.213786515 | 7.68E-116 | 0.45934826 | 1.66E-37 |
| *CDK2* | ENSG00000123374 | 1.171257421 | 4.77E-19 | 0.710757298 | 2.50E-12 |
| *MT2A* | ENSG00000125148 | 1.102052479 | 2.39E-197 | 1.643171582 | 0 |
| *HSPA2* | ENSG00000126803 | 1.081235784 | 1.46E-36 | 1.768061634 | 3.56E-74 |
| *ASIC3* | ENSG00000213199 | 1.080519984 | 0.000773754 | 1.808161353 | 3.53E-05 |
| *E2F3* | ENSG00000112242 | 0.967948498 | 2.75E-30 | 0.588297758 | 1.04E-16 |
| *ACTR1A* | ENSG00000138107 | 0.95672868 | 1.29E-47 | 0.440074269 | 2.78E-09 |
| *NUP85* | ENSG00000125450 | 0.942215278 | 2.32E-23 | 0.516421861 | 2.39E-09 |
| *RP2* | ENSG00000102218 | 0.931610215 | 1.49E-23 | 0.56112047 | 1.46E-07 |
| *MAN1A1* | ENSG00000111885 | 0.862264263 | 1.05E-10 | 1.42085316 | 1.21E-54 |
| *DDX11* | ENSG00000013573 | 0.861166096 | 5.40E-23 | 0.767985431 | 1.71E-27 |
| *CALM3* | ENSG00000160014 | 0.856848857 | 4.42E-127 | 0.504415974 | 3.25E-33 |
| *GNB4* | ENSG00000114450 | 0.844627408 | 9.30E-16 | 0.540419945 | 4.55E-13 |
| *PHC2* | ENSG00000134686 | 0.779111362 | 2.44E-38 | 0.502957493 | 3.00E-13 |
| *PSMD2* | ENSG00000175166 | 0.763404166 | 5.82E-137 | 0.385578491 | 5.64E-56 |
| *TUBB6* | ENSG00000176014 | 0.754105101 | 1.17E-29 | 0.785908547 | 2.41E-36 |
| *UBE2D1* | ENSG00000072401 | 0.716824358 | 2.96E-17 | 0.392037896 | 1.97E-05 |
| *PLAA* | ENSG00000137055 | 0.71319324 | 1.37E-16 | 0.491155937 | 2.96E-13 |
| *ATG16L1* | ENSG00000085978 | 0.709782584 | 5.03E-15 | 0.455236213 | 2.12E-06 |
| *HSPE1* | ENSG00000115541 | 0.700701424 | 4.10E-05 | 0.492299259 | 0.015405529 |
| *NPLOC4* | ENSG00000182446 | 0.687006198 | 2.52E-49 | 0.452715709 | 3.05E-25 |
| *PIK3R4* | ENSG00000196455 | 0.682749011 | 1.75E-15 | 0.622528514 | 1.32E-20 |
| *UFD1* | ENSG00000070010 | 0.629508242 | 1.07E-12 | 0.462753648 | 4.46E-09 |
| *FKBP4* | ENSG00000004478 | 0.586596378 | 9.26E-33 | 0.634200839 | 6.83E-46 |
| *HSPA1B* | ENSG00000204388 | 0.581239197 | 1.05E-30 | 1.524160326 | 2.77E-133 |
| *NCF4* | ENSG00000100365 | 0.572579904 | 0.008805443 | 1.30001445 | 0.001804568 |
| *HSPB8* | ENSG00000152137 | 0.554391121 | 0.00386002 | 0.975512411 | 6.70E-15 |
| *PSMD11* | ENSG00000108671 | 0.551338984 | 2.57E-32 | 0.39911055 | 2.80E-26 |
| *TPP1* | ENSG00000166340 | 0.546135988 | 1.32E-13 | 0.616735283 | 1.03E-18 |
| *BAG3* | ENSG00000151929 | 0.540836843 | 7.67E-16 | 0.743997455 | 6.53E-40 |
| *HMOX1* | ENSG00000100292 | 0.518641097 | 5.16E-06 | 1.306246404 | 5.57E-24 |
| *TMBIM6* | ENSG00000139644 | 0.51757618 | 2.05E-248 | 0.3788545 | 2.31E-118 |
| *SDF2* | ENSG00000132581 | 0.505220581 | 3.84E-07 | 0.532820052 | 5.28E-06 |
| *ADCY3* | ENSG00000138031 | 0.502215549 | 1.50E-05 | 0.519392574 | 8.33E-11 |
| *PLA2G6* | ENSG00000184381 | 0.498877842 | 6.02E-06 | 0.567728377 | 0.001349198 |
| *CALM1* | ENSG00000198668 | 0.485728604 | 7.31E-53 | 0.544282571 | 7.54E-76 |
| *LAMTOR3* | ENSG00000109270 | 0.477999112 | 1.75E-06 | 0.42113823 | 5.61E-05 |
| *PSMD13* | ENSG00000185627 | 0.458543734 | 1.13E-12 | 0.390247093 | 8.69E-11 |
| *TM7SF3* | ENSG00000064115 | 0.455884346 | 1.70E-14 | 0.487579342 | 4.62E-24 |
| *LGMN* | ENSG00000100600 | 0.404396425 | 8.89E-10 | 0.901774404 | 2.49E-56 |
| *HSPA1A* | ENSG00000204389 | 0.40206552 | 2.64E-14 | 1.759425687 | 1.21E-91 |
| **Downregulated** | | | | | |
| *FGF21* | ENSG00000105550 | -4.924302399 | 4.13E-08 | -4.361763648 | 3.39E-47 |
| *CLGN* | ENSG00000153132 | -4.826542351 | 2.56E-64 | -2.024728661 | 1.62E-07 |
| *ASNS* | ENSG00000070669 | -4.314248918 | 9.34E-10 | -2.303346962 | 4.41E-17 |
| *TUBB2B* | ENSG00000137285 | -4.174845861 | 2.29E-16 | -0.748231995 | 0.006465849 |
| *CHAC1* | ENSG00000128965 | -4.126526038 | 7.87E-81 | -2.903913065 | 2.06E-86 |
| *DDIT3* | ENSG00000175197 | -4.055146329 | 1.14E-277 | -3.631822503 | 2.01E-293 |
| *HSPA5* | ENSG00000044574 | -3.923123257 | 0 | -2.542568036 | 0 |
| *STC2* | ENSG00000113739 | -3.334885123 | 0 | -0.447775367 | 7.09E-60 |
| *SDF2L1* | ENSG00000128228 | -3.178724852 | 0 | -2.82853231 | 1.67E-219 |
| *HERPUD1* | ENSG00000051108 | -3.112125094 | 0 | -3.95005724 | 0 |
| *CXCL3* | ENSG00000163734 | -3.035947755 | 2.29E-09 | -2.854803659 | 5.28E-33 |
| *CXCL2* | ENSG00000081041 | -3.007769222 | 5.74E-30 | -1.79988476 | 1.77E-37 |
| *TRIB3* | ENSG00000101255 | -2.986570383 | 0 | -2.630213204 | 0 |
| *HYOU1* | ENSG00000149428 | -2.918206979 | 0 | -1.391210728 | 0 |
| *PDIA4* | ENSG00000155660 | -2.720220713 | 0 | -1.225931845 | 0 |
| *NUPR1* | ENSG00000176046 | -2.564733873 | 0 | -1.554700681 | 5.56E-223 |
| *HSP90B1* | ENSG00000166598 | -2.436002631 | 0 | -0.840599364 | 0 |
| *ADRB2* | ENSG00000169252 | -2.414931542 | 1.37E-52 | -1.194084087 | 1.08E-34 |
| *MANF* | ENSG00000145050 | -2.395961887 | 0 | -1.270002192 | 4.14E-116 |
| *ERO1B* | ENSG00000086619 | -2.31362125 | 3.51E-148 | -1.682920337 | 6.32E-73 |
| *AGR2* | ENSG00000106541 | -2.16803672 | 0 | -4.077315936 | 0 |
| *SEC24D* | ENSG00000150961 | -2.048443913 | 1.49E-177 | -1.301348702 | 1.22E-80 |
| *SESN2* | ENSG00000130766 | -1.93073381 | 1.15E-112 | -2.464210917 | 2.20E-215 |
| *DNAJB11* | ENSG00000090520 | -1.866865794 | 3.54E-186 | -1.085237493 | 1.38E-89 |
| *FICD* | ENSG00000198855 | -1.81525964 | 1.27E-12 | -3.309533689 | 5.72E-43 |
| *WIPI1* | ENSG00000070540 | -1.753798816 | 5.64E-26 | -0.737272783 | 0.001228393 |
| *DNAJB9* | ENSG00000128590 | -1.738369522 | 2.26E-64 | -2.866332369 | 1.75E-178 |
| *ERN1* | ENSG00000178607 | -1.679028895 | 4.54E-54 | -1.443710946 | 6.45E-59 |
| *SGTB* | ENSG00000197860 | -1.662296898 | 4.97E-23 | -0.734464201 | 1.73E-08 |
| *SEL1L* | ENSG00000071537 | -1.644003679 | 3.52E-291 | -1.664533649 | 0 |
| *CALR* | ENSG00000179218 | -1.621594472 | 0 | -0.584829439 | 1.07E-234 |
| *CBX4* | ENSG00000141582 | -1.500546445 | 1.30E-204 | -2.109216356 | 5.11E-240 |
| *CREB3L2* | ENSG00000182158 | -1.432492337 | 8.92E-51 | -1.875550397 | 1.56E-121 |
| *DNAJC3* | ENSG00000102580 | -1.421878241 | 3.79E-111 | -2.108160606 | 0 |
| *CEBPG* | ENSG00000153879 | -1.364041842 | 2.75E-94 | -1.154236534 | 1.46E-87 |
| *SYVN1* | ENSG00000162298 | -1.34238249 | 1.81E-151 | -1.693101366 | 6.85E-260 |
| *GFPT1* | ENSG00000198380 | -1.276300468 | 0 | -1.153625814 | 3.43E-290 |
| *XBP1* | ENSG00000100219 | -1.243509633 | 3.54E-185 | -1.669013621 | 0 |
| *GCLC* | ENSG00000001084 | -1.207839916 | 8.88E-215 | -1.901037073 | 0 |
| *TSPYL2* | ENSG00000184205 | -1.186615638 | 1.33E-28 | -1.425918788 | 1.36E-46 |
| *EDEM1* | ENSG00000134109 | -1.050306124 | 5.56E-89 | -0.394756012 | 1.57E-23 |
| *SERP1* | ENSG00000120742 | -0.993372259 | 3.82E-216 | -0.774029398 | 1.88E-100 |
| *GRPEL2* | ENSG00000164284 | -0.989078405 | 7.70E-31 | -0.710967887 | 7.01E-27 |
| *CHMP4C* | ENSG00000164695 | -0.957545058 | 6.15E-13 | -0.694700067 | 4.27E-08 |
| *SEC61A1* | ENSG00000058262 | -0.932474837 | 0 | -0.744352661 | 6.71E-171 |
| *HSPA9* | ENSG00000113013 | -0.914966812 | 0 | -0.596786716 | 1.54E-266 |
| *NCCRP1* | ENSG00000188505 | -0.906450067 | 1.71E-09 | -0.390384326 | 0.037388705 |
| *DAB2IP* | ENSG00000136848 | -0.878402862 | 1.07E-70 | -0.452789738 | 1.62E-14 |
| *UBE2J1* | ENSG00000198833 | -0.862507996 | 5.50E-57 | -1.042695207 | 2.11E-90 |
| *ATF4* | ENSG00000128272 | -0.834652476 | 0 | -0.451656596 | 1.79E-98 |
| *GABARAPL1* | ENSG00000139112 | -0.828119414 | 1.38E-82 | -0.728350687 | 5.00E-55 |
| *SELENOS* | ENSG00000131871 | -0.816125304 | 1.65E-49 | -0.579681843 | 9.15E-24 |
| *SEC63* | ENSG00000025796 | -0.802668214 | 3.69E-102 | -0.588456207 | 2.37E-63 |
| *PLAC8* | ENSG00000145287 | -0.712388882 | 1.12E-23 | -1.293975128 | 1.16E-90 |
| *FKBP14* | ENSG00000106080 | -0.681915524 | 2.93E-15 | -0.635261332 | 5.93E-18 |
| *ATF6* | ENSG00000118217 | -0.668985907 | 5.42E-40 | -0.565396967 | 1.48E-34 |
| *FBXO27* | ENSG00000161243 | -0.656284586 | 7.22E-12 | -0.732813135 | 5.54E-16 |
| *DERL2* | ENSG00000072849 | -0.625373583 | 1.23E-21 | -1.345918703 | 5.16E-84 |
| *CAMK2D* | ENSG00000145349 | -0.620012443 | 7.15E-18 | -0.429820377 | 2.95E-10 |
| *EIF2AK3* | ENSG00000172071 | -0.613109202 | 1.91E-06 | -1.431533926 | 1.70E-49 |
| *NFE2L1* | ENSG00000082641 | -0.603423397 | 9.30E-152 | -0.568852262 | 5.93E-95 |
| *SERPINH1* | ENSG00000149257 | -0.578331304 | 5.82E-68 | -0.841884954 | 1.33E-117 |
| *EIF2S2* | ENSG00000125977 | -0.566661969 | 6.61E-74 | -0.573339186 | 1.14E-96 |
| *ZFAND1* | ENSG00000104231 | -0.56244554 | 1.10E-16 | -1.29354718 | 2.28E-74 |
| *ESR1* | ENSG00000091831 | -0.537129875 | 4.46E-13 | -0.766104564 | 6.68E-28 |
| *SELENOK* | ENSG00000113811 | -0.530167188 | 1.98E-07 | -0.704763966 | 1.50E-14 |
| *CTSB* | ENSG00000164733 | -0.526393529 | 1.25E-299 | -0.398334346 | 6.08E-192 |
| *RING1* | ENSG00000204227 | -0.511448564 | 1.10E-17 | -0.506103005 | 4.88E-10 |
| *CDK6* | ENSG00000105810 | -0.509721725 | 4.87E-11 | -0.389557458 | 1.28E-16 |
| *EGLN2* | ENSG00000269858 | -0.461459346 | 0.003762406 | -0.792742691 | 6.40E-06 |
| *NUDT2* | ENSG00000164978 | -0.425863791 | 0.000384185 | -0.873762877 | 1.82E-10 |
| *ENTPD5* | ENSG00000187097 | -0.425530515 | 2.16E-05 | -0.598384685 | 9.87E-15 |
| *NFKB1* | ENSG00000109320 | -0.410999432 | 2.85E-13 | -0.471993322 | 3.46E-24 |
| *SLC9A1* | ENSG00000090020 | -0.395690753 | 2.26E-14 | -0.431496269 | 3.71E-13 |

**Table S5. List of the primer pairs used for quantitative real-time PCR analysis**

| **Primer name** | **Primer sequence (5’🡪3’)** |
| --- | --- |
| hLSD1-RT-F | ATGGATGTCACACTTTTGGA |
| hLSD1-RT-R | CCAAGATCAGCTACATAGTTTC |
| hG9a-RT-F | AAGCACATCGAGGTGATCCG |
| hG9a-RT-R | CAGATGTTCTCCTCGTTGTCAG |
| hChop-RT-F | GGAACCTGAGGAGAGAGTGTTC |
| hChop-RT-R | CAAGCTCCATGTAGCAAACAGT |
| hBip-RT-F | CATCAACGAGCCTACGGCAG |
| hBip-RT-R | TGGCCACAACTTCGAAGACA |
| hIRE1-RT-F | CGGCCTCGGGATTTTTGGAA |
| hIRE1-RT-R | CCTGCAGGACTGGATCTTCT |
| hATF4-RT-F | TCCAACAACAGCAAGGAGGAT |
| hATF4-RT-R | TCCAACGTGGTCAGAAGGTC |
| hPERK-RT-F | ATGAGACAGAGTTGCGACCG |
| hPERK-RT-R | TGGATGACACCAAGGAACCG |
| hATF6-RT-F | AACCCGTATTCTTCAGGGTGC |
| hATF6-RT-R | ACTCCCTGAGTTCCTGCTGATA |
| hXBP1us-RT-F | TGCCAGAGATCGAAAGAAGGC |
| hXBP1us-RT-R | CCAAGCGCTGTCTTAACTCCT |
| hXBP1s-RT-F | TGCTGAGTCCGCAGCAGGTG |
| hXBP1s-RT-R | GCTGGCAGGCTCTGGGGAAG |
| h-DNAJB9-RT-F | ATAAAAGCCCTGATGCTGAAGC |
| h-DNAJB9-RT-R | GCCATTGGTAAAAGCACTGTGT |

**Table S6. List of target-specific shRNA hairpin sequences for knocking down human *LSD1* (hLSD1) and *G9a* (hG9a)**

| **shRNA construct** | **Sequence (5’🡪3’)** |
| --- | --- |
| hLSD1-shRNA-1 | CCGGGCCTAGACATTAAACTGAATACTCGAGTATTCAGTTTAATGTCTAGGCTTTTTG |
| hLSD1-shRNA-2 | CCGGGCTCCAATACTGTTGGCACTACTCGAGTAGTGCCAACAGTATTGGAGCTTTTTG |
| hLSD1-shRNA-3 | CCGGCCAACAATTAGAAGCACCTTACTCGAGTAAGGTGCTTCTAATTGTTGGTTTTTG |
| hLSD1-shRNA-4 | CCGGGCTACATCTTACCTTAGTCATCTCGAGATGACTAAGGTAAGATGTAGCTTTTTG |
| hG9a-shRNA-1 | CCGGCCTCTTCGACTTAGACAACAACTCGAGTTGTTGTCTAAGTCGAAGAGGTTTTTG |
| hG9a-shRNA-2 | CCGGGCTCCAGGAATTTAACAAGATCTCGAGATCTTGTTAAATTCCTGGAGCTTTTTG |
| hG9a-shRNA-3 | CCGGCGAGAGAGTTCATGGCTCTTTCTCGAGAAAGAGCCATGAACTCTCTCGTTTTTG |
| hG9a-shRNA-4 | CCGGCTCCAGGAATTTAACAAGATTCTCGAGAATCTTGTTAAATTCCTGGAGTTTTTG |
