# Supplementary material for: Targeting the LSD1-G9a-ER Stress Pathway as a Novel Therapeutic Strategy for Esophageal Squamous Cell Carcinoma
Source: Research (Wash D C). 2022 Jun 1;2022:9814652. doi: 10.34133/2022/9814652 (PMC9185438; doi:10.34133/2022/9814652)
Supplement: Supplementary Materials — Figure S1: inhibiting both LSD1 and G9a significantly reduces the viability of esophageal cancer cells. Figure S2: genetically silencing LSD1 and G9a increases ESCCs cell death. Figure S3: systemic treatment with doxycycline or inhibitors of LSD1 and/or G9a does not affect the body weight of mice bearing tumors derived from xenografted esophageal cancer cells. Figure S4: LSD1 and G9a expression is upregulated in esophageal cancer tissues obtained from The Cancer Genome Atlas database. Figure S5: Gene Set Enrichment Analysis of RNA-seq data in ESCCs following knockdown and pharmacological inhibition of LSD1 alone, G9a alone, or both LSD1 and G9a. Figure S6: targeting LSD1 and/or G9a induces S-phase arrest and apoptosis in ESCCs. Figure S7: inhibitors of ER-stress sensitize esophageal cancer cells to pharmacological inhibitors of LSD1 and G9a. Figure S8: targeting modulators of ER-stress modulators sensitizes ESCCs to the effects of inhibiting LSD1 and G9a in vivo. Table S1: list of the chemicals and compounds used in this study. Table S2: clinicopathological information of all 114 patients with esophageal squamous cell carcinoma. Table S3: univariate and multivariate analyses of the factors correlated with overall survival of ESCC patients. Table S4: list of genes that were significantly upregulated or downregulated (defined as a fold change in either direction ≥ 1.3 and p < 0.05) in ESCCs in response to genetic silencing and inhibition of G9a. Table S5: list of the primer pairs used for quantitative real-time PCR analysis. Table S6: list of target-specific shRNA hairpin sequences for knocking down human LSD1 (hLSD1) and G9a (hG9a). [file 9814652.f1.zip › Supplementary Materials_Final.doc]

**Supplementary Materials**

**Figure S1**. Inhibiting both LSD1 and G9a significantly reduces the viability of esophageal cancer cells.

**Figure S2.** Genetically silencing LSD1 and G9a increases ESCCs cell death.

**Figure S3.** Systemic treatment with doxycycline or inhibitors of LSD1 and/or G9a does not affect the body weight of mice bearing tumors derived from xenografted esophageal cancer cells.

**Figure S4.** LSD1 and G9a expression is upregulated in esophageal cancer tissues obtained from The Cancer Genome Atlas database.

**Figure S5.** Gene Set Enrichment Analysis of RNA-seq data in ESCCs following knockdown and pharmacological inhibition of LSD1 alone, G9a alone, or both LSD1 and G9a.

**Figure S6.** Targeting LSD1 and/or G9a induces S-phase arrest and apoptosis in ESCCs.

**Figure S7.** Inhibitors of ER-stress sensitize esophageal cancer cells to pharmacological inhibitors of LSD1 and G9a.

**Figure S8.** Targeting modulators of ER-stress modulators sensitizes ESCCs to the effects of inhibiting LSD1 and G9a *in vivo.*

**Table S1.** List of the chemicals and compounds used in this study.

**Table S2.** Clinicopathological information of all 114 patients with esophageal squamous cell carcinoma.

**Table S3.** Univariate and multivariate analyses of the factors correlated with overall survival of ESCC patients.

**Table S4.** List of genes that were significantly upregulated or downregulated (defined as a fold change in either direction ≥1.3 and *p*<0.05) in ESCCs in response to genetic silencing and inhibition of G9a.

**Table S5**. List of the primer pairs used for quantitative real-time PCR analysis.

**Table S6**. List of target-specific shRNA hairpin sequences for knocking down human *LSD1* (hLSD1) and *G9a* (hG9a).
